# A GPS2-like protein interacts with HOS15 and HDA6 to form a repressor complex that regulates ABA signaling and drought adaptation in *Arabidopsis*

**DOI:** 10.1016/j.xplc.2026.101843

**Published:** 2026-04-03

**Authors:** Akhtar Ali, Shah Zareen, Zein Eddin Bader, Junghoon Park, Irfan Ullah Khan, Kisuk Park, Nassem Albakri, Min Jae Bae, Ray A. Bressan, Jose M. Pardo, Dae-Jin Yun, Zheng-Yi Xu

**Affiliations:** 1 School of Advanced Biotechnology, Global Plant Stress Research Center, Konkuk University, Seoul 05029, Korea; 2 Department of Molecular Stress Physiology, Center of Plant Systems Biology and Biotechnology, Plovdiv, Bulgaria; 3 Institute of Natural Medicine, University of Toyama, Toyama 930-0194, Japan; 4 School of Biological Sciences, Seoul National University, Seoul 08826, Korea; 5 Department of Horticulture and Landscape Architecture, Purdue University, 625 Agriculture Mall Dr., West Lafayette, IN 47907-2010, USA; 6 Instituto de Bioquimica Vegetal y Fotosintesis, cicCartuja, CSIC-Universidad de Sevilla, Americo Vespucio 49, 41092 Sevilla, Spain; 7 Key Laboratory of Molecular Epigenetics of the Ministry of Education (MOE), Northeast Normal University, Changchun, China

**Keywords:** chromatin remodeling, histone acetylation/methylation, ABA signaling, GPL, HOS15, HDA6

## Abstract

Plants rely on chromatin-mediated transcriptional control to fine-tune stress responses; however, the evolutionary conservation and functional diversification of repressor complexes remain incompletely understood. Here, we identify GPS2-like protein (GPL) as the missing component of the plant counterpart of the animal nuclear receptor–corepressor complex. GPL interacts with HOS15, PWR, and HDA6/HDA9 to form a chromatin repressor module that suppresses abscisic acid (ABA)-responsive genes. Loss-of-function *gpl* mutants exhibit ABA hypersensitivity and enhanced drought tolerance, whereas *GPL* overexpression confers ABA insensitivity. Mechanistically, GPL promotes histone H3K9 deacetylation and dimethylation at stress-responsive loci and stabilizes HOS15 to ensure its nuclear retention. Global RNA sequencing reveals widespread derepression of ABA-responsive transcriptional networks in *gpl* mutants. Under stress conditions, ABA destabilizes the GPL–HOS15 complex, relieving repression and activating defense-related genes. These findings establish GPL as the plant homolog of GPS2 and reveal that the GPL–HOS15 repressor complex functions as a chromatin-mediated rheostat to dynamically balance growth and drought adaptation. Our work provides mechanistic insight into stress-responsive chromatin remodeling and identifies GPL as a potential target for engineering climate-resilient crops.

## Teaser

This study identifies GPL as a plant GPS2-like component of the HOS15-associated corepressor complex that negatively regulates abscisic acid (ABA) signaling and drought adaptation in *Arabidopsis*. GPL interacts with HOS15 and HDA6 to repress ABA-responsive genes through chromatin modification. Loss of *GPL* enhances ABA sensitivity and drought tolerance by derepressing stress-responsive transcriptional networks.

## Introduction

Plants, as sessile organisms, must dynamically adapt their growth, development, and physiology to fluctuating environmental conditions, including abiotic stresses such as drought, salinity, and temperature extremes, as well as biotic challenges such as pathogen attack (Oh et al., 2012; Wu et al., 2012; Zhu, 2016). These adaptive responses are regulated by intricate molecular mechanisms, with plant hormones playing central roles in coordinating biochemical and physiological processes (Verma et al., 2016; Waadt et al., 2022). Among these hormones, abscisic acid (ABA) acts as a key regulator of stress responses and developmental programs, including seed maturation, embryo morphogenesis, stomatal movement, and flowering time (Cao et al., 2011; Collin and Daszkowska-Golec, 2025; Wei et al., 2025). Elucidating the molecular machinery underlying ABA-mediated stress adaptation is therefore essential for developing climate-resilient crops, a pressing need in the face of global environmental challenges (Rivero et al., 2022; Xiong et al., 2022).

Epigenetic regulation, including chromatin remodeling and histone modification, is a key mechanism by which plants fine-tune gene expression in response to environmental cues (Kim et al., 2015; Asensi-Fabado et al., 2017; Tresas et al., 2025). In *Arabidopsis*, the WD40-repeat protein HOS15 functions as a substrate receptor of the DDB1–CUL4 E3 ubiquitin ligase complex, targeting key regulators such as OPEN STOMATA 1 (OST1) and DROUGHT-INDUCED LIKE 19 (DIL9) for degradation, thereby negatively modulating ABA signaling and drought responses (Ali et al., 2019; Zareen et al., 2025). Beyond its role in protein turnover, HOS15 forms multifunctional complexes with POWERDRESS (PWR) and HISTONE DEACETYLASE 9 (HDA9), contributing to chromatin remodeling and transcriptional reprogramming in processes such as stress adaptation, pathogen defense, flowering-time regulation, and microRNA biogenesis (Park et al., 2018a, 2019, 2023; Suzuki et al., 2018; Ali and Yun, 2020; Lim et al., 2021, 2023; Zareen et al., 2022). In addition, HOS15 interacts with HISTONE DEACETYLASE 2C (HD2C) to regulate cold stress responses, NON-EXPRESSOR OF PR1 (NPR1) to modulate immunity, and GIGANTEA (GI) to control flowering, underscoring its central and versatile role in plant regulatory networks (Park et al., 2018a; Shen et al., 2020; Ahn et al., 2023).

In animal systems, the nuclear receptor (NR)–corepressor complex—comprising NCoR1, HDAC3, TBL1, and GPS2 (G-protein Pathway Suppressor 2)—regulates gene expression across diverse physiological processes, including metabolism, cell proliferation, DNA repair, apoptosis, brain development, and breast cancer (Zhang et al., 2002; Yoon, 2003; Rosenfeld et al., 2006; Karagianni and Wong, 2007; Huang et al., 2015; Ferrero et al., 2025). Although plant homologs of NCoR1 (PWR), HDAC3 (HDA9), and TBL1 (HOS15) have been identified (Park et al., 2018a; Mayer et al., 2019; Lim et al., 2021), the plant counterpart of GPS2 has remained unknown, limiting our understanding of the evolutionary conservation and functional adaptation of this repressor complex in plants.

Here, we identify GPS2-like (GPL; AT3G47850) as a novel HOS15-interacting protein homologous to human GPS2, thereby completing the plant equivalent of the NR–corepressor complex. Our findings reveal that GPL and HOS15 exhibit interdependent protein stability, as loss of either protein leads to destabilization of the other. Similar to *hos15-2* mutants, *gpl* loss-of-function mutants display severe growth defects, hypersensitivity to ABA and salt stress, and enhanced drought resistance, indicating that GPL and HOS15 function within a shared regulatory pathway. Global RNA sequencing (RNA-seq) analyses demonstrate that GPL represses ABA-responsive genes, such as *AHG3*, *RD20*, and *RD29A*, through epigenetic mechanisms, as evidenced by reduced DNA methylation and increased histone H3K9 acetylation in *gpl* mutants. These results establish the GPL–HOS15 complex as a dynamic epigenetic rheostat that modulates ABA signaling and drought responses, offering insight into transcriptional reprogramming during plant stress adaptation and potential avenues for engineering stress-resilient crops.

## Results

### GPL, a novel HOS15 interactor and plant homolog of GPS2

**Figure 1:**
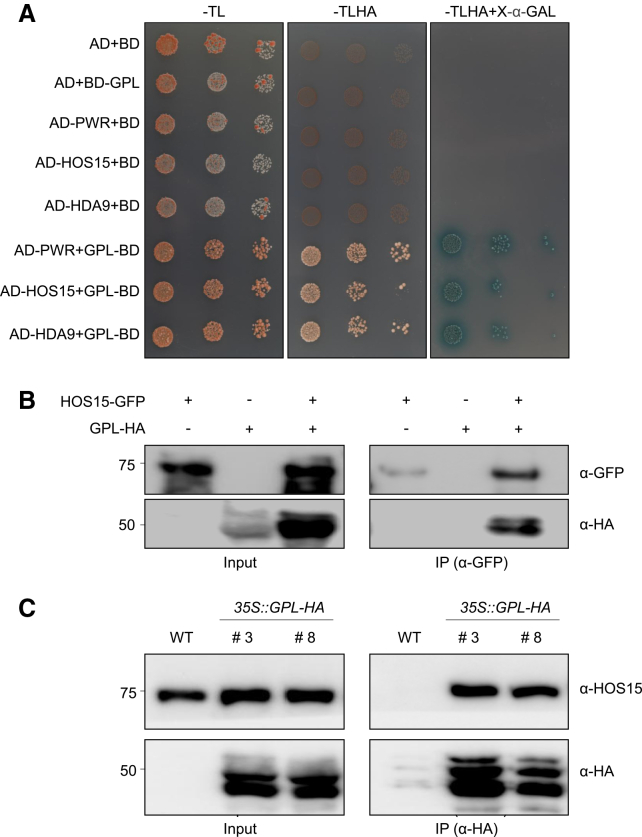
GPL interacts with HOS15, HDA9, and PWR. **(A)** GPL physically interacts with PWR, HOS15, and HDA9. A Y2H assay was performed using HOS15, PWR, and HDA9 as prey and GPL as bait to assess their interactions. Empty vectors (AD or BD) were used as negative controls. Yeast cells were grown for 5 days on media supplemented with X-α-GAL but lacking TL, TLHA, or TLHA, after which images were taken. **(B)** GPL interacts with HOS15 as shown by co-immunoprecipitation. GPL-HA and HOS15-GFP were transiently co-expressed in tobacco leaves. After 3 days, total proteins were extracted from leaves and immunoprecipitated with anti-GFP antibodies. Immunoblot analysis was performed using anti-GFP and anti-HA antibodies. **(C)** GPL interacts with HOS15 in *Arabidopsis*. Total proteins were extracted from 10-day-old *35S*::*GPL-HA Arabidopsis* seedlings and immunoprecipitated with anti-HA antibodies. Immunoblot analysis was performed using anti-HA and anti-HOS15 antibodies. WT plants were used as a control.

HOS15, a WD40-repeat protein, is a key regulator of ABA signaling and drought stress responses. It functions as a substrate receptor of the DDB1–CUL4 E3 ubiquitin ligase complex, targeting proteins such as OST1 for degradation (Ali et al., 2019). Additionally, HOS15 forms a transcriptional corepressor complex with PWR and HDA9, modulating chromatin structure to regulate gene expression during stress adaptation, flowering, and other developmental processes (Suzuki et al., 2018; Mayer et al., 2019; Zareen et al., 2022; Lim et al., 2023). To further investigate the HOS15 regulatory network, we reanalyzed our previously reported HOS15 immunoprecipitation–mass spectrometry (IP–MS) dataset to identify novel interacting partners (Park et al., 2018b). Among the identified proteins, AT3G47850 stood out due to its 26.7% sequence similarity and structural resemblance to human GPS2, a core component of the animal NR–corepressor complex (Supplemental Figures 1 and 2; Zhang et al., 2002; Huang et al., 2015). We designated this protein GPS2-like (GPL). Phylogenetic analysis revealed that GPL is conserved as a single-copy gene across plant species, underscoring its evolutionary significance (Supplemental Figure 3 and Supplemental Table 1). In animals, GPS2 interacts with NCoR1, HDAC3, and TBL1 to form the NR–corepressor complex, which represses gene expression in processes such as metabolism and cell proliferation (Zhang et al., 2002; Yoon, 2003; Ferrero et al., 2025). To test whether GPL functions within a similar complex in plants, we investigated its interactions with the *Arabidopsis* homologs of these components: HOS15 (homolog of TBL1), PWR (homolog of NCoR1), and HDA9 (homolog of HDAC3). Yeast two-hybrid (Y2H) assays confirmed that GPL physically interacts with HOS15, PWR, and HDA9 (Figure 1A). The interaction between HOS15 and GPL was further validated by co-immunoprecipitation (co-IP) assays in both transient expression systems and stable *Arabidopsis* transgenic lines (Figures 1B and 1C). Furthermore, IP–MS analysis reconfirmed that GPL interacts with HOS15, PWR, histone deacetylases HDA9 and HDA6, RPN1A, a 26S proteasome regulatory subunit, and several other proteins, including transcription factors, highlighting the involvement of GPL in epigenetic regulation (Supplemental Figure 4 and Supplemental Table 2). These findings establish GPL as the plant homolog of GPS2 and suggest that it forms a corepressor complex with HOS15, PWR, and HDA9/HDA6. This complex likely plays a critical role in the epigenetic regulation of target loci, thereby bridging plant and animal chromatin remodeling pathways and expanding our understanding of HOS15’s multifaceted regulatory functions.

### Loss-of-function *gpl* mutants display ABA hypersensitivity and enhanced drought resilience

Our previous studies have shown that *hos15-2* mutants exhibit ABA hypersensitivity and enhanced drought resistance, whereas *pwr* and *hda9* mutants display ABA insensitivity and drought sensitivity (Ali et al., 2019; Baek et al., 2020; Khan et al., 2020). Given that GPL interacts with HOS15, PWR, and HDA9 within a plant NR–corepressor complex analog (Figure 1A and Supplemental Table 2), we hypothesized that GPL also plays an important role in regulating ABA signaling and stress responses. To test this hypothesis, we generated *gpl* loss-of-function mutants using the CRISPR–Cas9 system by targeting the *GPL* gene (AT3G47850) (Supplemental Figure 3A). The mutants were validated by genotyping and sequencing to confirm targeted disruptions (Supplemental Figures 4B and 3C). Phenotypic analysis revealed that *gpl* mutants exhibited dwarfism and early flowering, resembling the *hos15-2*, *pwr*, and *hda9* mutants (Supplemental Figures 5D–5G; Lim et al., 2023). Notably, overexpression of *GPL* in the *gpl-1* mutant background fully rescued these phenotypic defects (Supplemental Figures 5F–5I).

**Figure 2:**
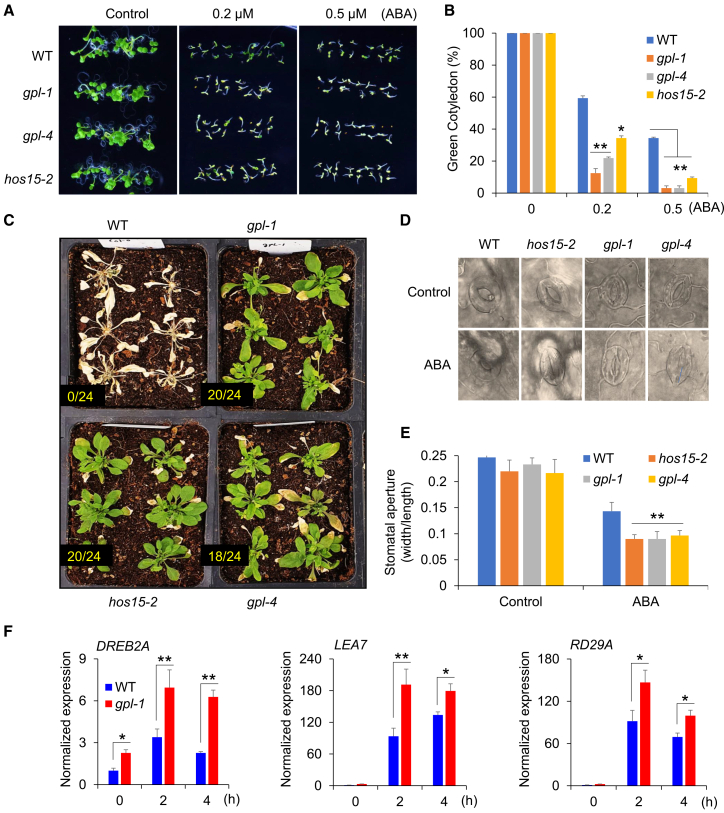
*GPL*-CRISPR lines are ABA hypersensitive and drought resilient. **(A)** The *GPL* mutation confers ABA hypersensitivity during germination. Seeds of WT (Col-0), *hos15-2*, and *gpl* mutants were germinated on ½ MS medium supplemented with the indicated concentrations of ABA (μM) in a long-day chamber at 22°C. Photographs were taken on day 6 (control) and day 10 (ABA) after germination. The *hos15-2* mutant was used as an experimental control. **(B)** Green cotyledons were counted after 7 days. Error bars represent SE (*n* = 3 independent experiments, each with three replicates). Statistical significance was determined by Student’s *t-*test (∗*p* < 0.05, ∗∗*p* < 0.01). **(C)***gpl* loss-of-function mutants exhibit enhanced tolerance to dehydration stress. Seeds of WT, *hos15-2*, and *gpl* mutants were germinated on ½ MS medium for 1 week and then transferred to soil. Drought-tolerance assays were performed on 3-week-old plants by withholding water for 14 days followed by rewatering. Photographs were taken 3 days after rewatering. The *hos15-2* mutant served as the control. Survival rates were measured using two pots per genotype after the drought treatment. **(D)***gpl* mutants exhibit rapid stomatal closure upon exposure to ABA (10 μM). The *hos15-2* mutant was used as a positive control. **(E)** Stomatal size (width/length) was measured using ImageJ software, with error bars representing SE (*n* = 10 stomata per genotype). Statistical significance was determined by Student’s *t-*test (∗∗*p* < 0.01). **(F)** GPL represses ABA- and drought-responsive genes. Expression of stress-related genes was analyzed in WT and *gpl-1* mutant plants. Seeds of WT and *gpl-1* were grown on ½ MS medium for 7 days and then treated with 50 μM ABA at room temperature for 0, 2, and 4 h. After treatment, total RNA was extracted, and RT–qPCR analysis was performed. *UBQ5* was used as the internal control. Error bars represent SE. Statistical significance was determined by Student’s *t-*test (∗*p* < 0.05, ∗∗*p* < 0.01).

To assess the role of GPL in ABA signaling, we evaluated *gpl* mutants for ABA sensitivity during germination and root growth. Compared with wild-type (WT) Columbia-0 (Col-0) plants, *gpl* mutants displayed pronounced ABA hypersensitivity, as indicated by reduced germination rates and inhibited root elongation under ABA treatment (Figures 2A and 2B; Supplemental Figure 6). In contrast, GPL overexpression lines were insensitive to ABA treatment during germination, supporting the role of GPL as a negative regulator (Supplemental Figures 7A and 7B). To further examine the role of GPL in drought stress, we subjected *gpl* mutants to a 14-day water-withholding assay followed by rewatering. The *gpl* mutants exhibited significantly greater drought tolerance than WT plants, with higher survival rates (Figure 2C), whereas GPL overexpression lines showed WT-like responses to dehydration (Supplemental Figure 7C). This drought resilience in *gpl* mutants was associated with enhanced stomatal closure in response to ABA, as observed in leaf epidermal fragments (Figures 2D and 2E; Supplemental Figures 7D and 7E), suggesting that more rapid stomatal closure contributes to their drought-tolerant phenotype.

To investigate the molecular basis underlying these phenotypes, we analyzed the expression of ABA- and drought-responsive genes in *gpl* mutants. Genes such as *DREB2A*, *LEA7*, and *RD29A* were significantly upregulated in *gpl* mutants compared with WT following ABA treatment (Figure 2F), supporting a role for GPL as a transcriptional repressor. These findings align with the ABA hypersensitivity and drought resistance observed in *hos15-2* mutants (Ali et al., 2019) but contrast with the ABA insensitivity and drought sensitivity of *pwr* and *hda9* mutants (Baek et al., 2020; Khan et al., 2020). Collectively, these results demonstrate that, similar to HOS15, GPL functions as a negative regulator of ABA signaling and enhances drought resilience, likely through its incorporation into the HOS15–PWR–HDA9/HDA6 repressor complex, with distinct contributions to stress-response pathways.

### Interdependent stability and ABA-mediated regulation of GPL and HOS15

**Figure 3:**
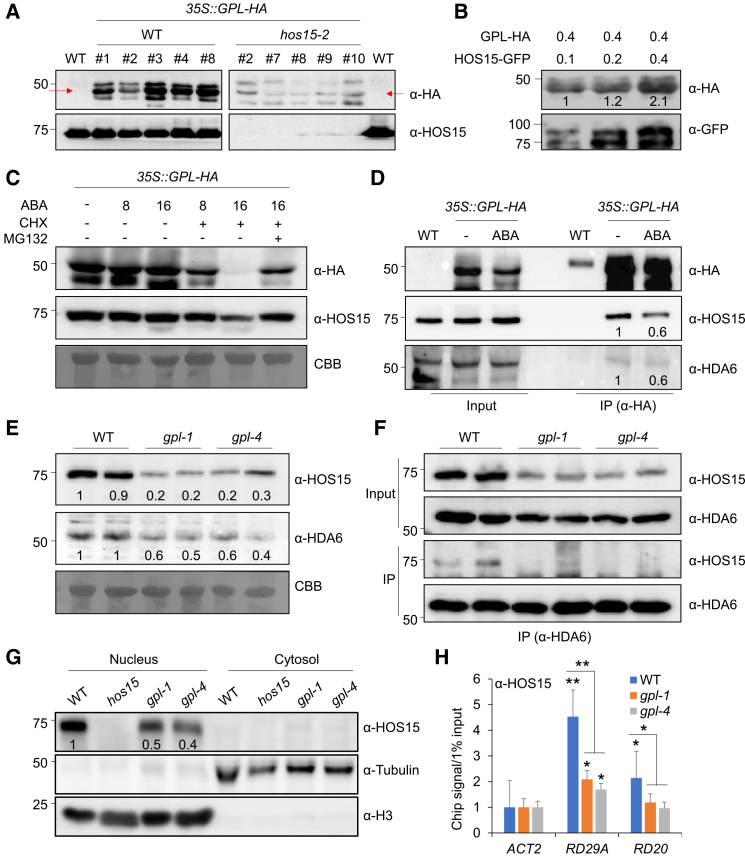
GPL and HOS15 reciprocally regulate each other’s stability. **(A)** HOS15 function is required for GPL stability. Total proteins were extracted from 10-day-old transgenic lines expressing *35S*::*GPL-HA* in WT and the *hos15-2* background. Immunoblot analysis was performed using anti-HA and anti-HOS15 antibodies. Red arrows indicate GPL-HA (numbers denote independent transgenic lines). **(B)** HOS15 promotes GPL protein accumulation. GPL and HOS15 were transiently expressed in tobacco leaves, with GPL kept constant (OD 0.4) while HOS15 levels were gradually increased. Total proteins were extracted and immunoblotted with anti-HA and anti-GFP antibodies. **(C)** GPL is destabilized upon ABA treatment. Total proteins were extracted from 10-day-old *35S*::*GPL-HA-*expressing *Arabidopsis* seedlings treated with ABA (50 μM) and CHX for the indicated times (h). Immunoblot analysis was performed using anti-HA and anti-HOS15 antibodies. Coomassie Brilliant Blue (CBB) staining was used as a loading control. **(D)** GPL interacts with HOS15 and HDA6 in *Arabidopsis*. Ten-day-old *35S*::*GPL-HA-*expressing *Arabidopsis* seedlings were treated with or without 50 μM ABA for 4 h. Total proteins were extracted and immunoprecipitated with anti-HA antibodies. Immunoblot analysis was performed using anti-HA, anti-HOS15, and anti-HDA6 antibodies. WT was used as a control. **(E)** HOS15 and HDA6 proteins are destabilized in *gpl* mutants. Total proteins were extracted from 10-day-old seedlings of WT and *gpl* mutants (two lanes indicate two biological replicates). Immunoblot analysis was performed using anti-HOS15 and anti-HDA6 antibodies. CBB staining was used as a loading control. **(F)** The interaction between HOS15 and HDA6 is impaired in *gpl* mutants. Total proteins were extracted from 10-day-old seedlings of WT and *gpl* mutants and immunoprecipitated with anti-HDA6 antibodies. Immunoblot analysis was performed using anti-HOS15 and anti-HDA6 antibodies (two lanes indicate two biological replicates). **(G)** GPL function is required for HOS15 nuclear retention. Total proteins were extracted from 10-day-old seedlings of WT (Col-0) and *gpl* mutants. Immunoblot analysis was performed using anti-HOS15 antibodies. Histone H3 and tubulin were used as nuclear and cytosolic loading controls, respectively. **(H)** The association of HOS15 with the *RD29A* and *RD20* promoters is reduced in *gpl* mutants. The promoter regions of *RD29A* and *RD20* were analyzed by ChIP–qPCR. ChIP assays were carried out using anti-HOS15 antibodies. ACTIN2 served as the internal control. Error bars represent SE. Statistical significance was determined by Student’s *t-*test (∗*p* < 0.05, ∗∗*p* < 0.01).

Our previous work demonstrated that HOS15 stability depends on functional PWR and HDA9, key components of a plant NR–corepressor complex analog (Lim et al., 2023). Given that GPL interacts with HOS15, PWR, and HDA9 (Figures 1A–1C), we hypothesized that GPL and HOS15 may also reciprocally regulate each other’s stability. To test this, we generated *35S*::*GPL-HA* transgenic lines in both WT and *hos15-2* backgrounds. Immunoblot analysis revealed significantly reduced GPL protein levels in *hos15-2* compared with WT, indicating that HOS15 is required for GPL stability (Figure 3A). Treatment with the proteasome inhibitor MG132 prevented GPL degradation, whereas the protein synthesis inhibitor cycloheximide (CHX) accelerated its decay, supporting proteasome-mediated regulation of GPL (Supplemental Figure 8). Conversely, increased HOS15 levels stabilized the GPL protein (Figure 3B). Together, these results identify HOS15 as a key determinant of GPL stability. Given their roles in ABA signaling, we next examined the effects of ABA on GPL and HOS15 stability (Ali et al., 2019). In 10-day-old *35S*::*GPL-HA* seedlings treated with ABA for 16 h in the presence of CHX, GPL protein was completely degraded, and HOS15 protein levels were similarly compromised (Figure 3C). This coordinated destabilization suggests that ABA triggers dissociation of the GPL–HOS15 complex, enabling rapid stress responses. Because HOS15 interacts with HISTONE DEACETYLASE 6 (HDA6) (Park et al., 2018a) and both *hos15-2* and *hda6* mutants exhibit ABA hypersensitivity (Chen et al., 2010; Luo et al., 2012; Ali et al., 2019), we next investigated whether GPL also interacts with HDA6. Co-IP using anti-HA antibodies in *35S*::*GPL-HA* seedlings confirmed that GPL associates with both HDA6 and HOS15, and ABA treatment partially weakened these interactions (Figure 3D). To further explore GPL’s role in stabilizing this complex, we examined HOS15 and HDA6 protein levels in *gpl* mutants. Both proteins were significantly reduced in *gpl* mutants compared with WT (Figure 3E). Moreover, the interaction between HOS15 and HDA6 was abolished in *gpl* mutants, indicating that GPL is essential for maintaining the integrity of this repressor complex (Figure 3F). Consistent with this, nuclear–cytoplasmic fractionation and chromatin immunoprecipitation (ChIP) assays revealed that both nuclear accumulation of HOS15 and its binding to the promoters of ABA-responsive genes (RD29A and RD20) were compromised in *gpl* mutants (Figures 3G and 3H). To distinguish effects on nuclear localization from those on protein stability, *gpl* mutants were treated with the proteasome inhibitor MG132 to restore HOS15 protein levels to those observed in WT plants. Indeed, MG132 treatment stabilized HOS15 in *gpl* mutants (Supplemental Figure 9A). However, ChIP–qPCR analyses revealed that, even when HOS15 abundance was restored to WT levels (*gpl* + MG132), its association with target loci (e.g., *RD29A*) remained reduced in *gpl* mutants (Supplemental Figure 9B). These findings demonstrate that GPL not only stabilizes HOS15 but also promotes its transcriptional regulatory activity, highlighting their interdependent roles in the epigenetic regulation of the ABA pathway.

### GPL negatively regulates the ABA-responsive transcriptional network

**Figure 4:**
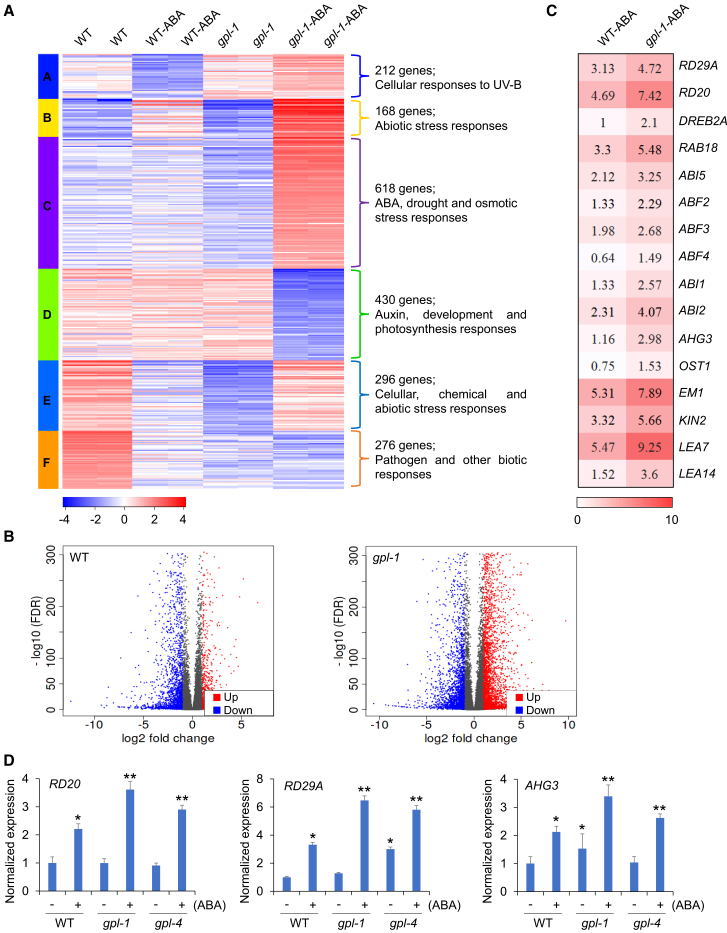
GPL represses ABA-responsive genes. **(A)** Heatmap of DEGs between WT and *gpl-1* mutants with or without ABA treatment. ABA- and abiotic stress-responsive genes are strongly upregulated in *gpl* mutants, whereas genes related to auxin signaling, growth and development, and pathogen and other biotic stress responses are downregulated. Expression values are shown as gene-wise *Z* scores (genes centered by subtracting the mean and scaled by the standard deviation, with values capped at ±3). Clusters “A” to “F” on the left border represent different clusters (see Supplemental Figure 10 for Gene Ontology terms associated with each cluster). **(B)** Volcano plot showing significantly upregulated and downregulated genes (FDR < 0.05) in WT and *gpl-1* plants upon ABA treatment. In WT plants, 3,003 DEGs were identified, including 986 (32.8%) upregulated and 2,017 (67.2%) downregulated genes. In *gpl-1* plants, 6,069 DEGs were identified, including 3,617 (59.6%) upregulated and 2,452 (40.4%) downregulated genes. **(C)** Heatmap of selected ABA-responsive genes in WT and *gpl-1* based on RNA-seq data. ABA-responsive genes were strongly upregulated in *gpl* mutants compared with WT (fold changes indicated). Color intensity represents log_2_-transformed fold-change values, with higher positive values shown in warmer colors and negative values in cooler colors. **(D)** GPL represses the expression of ABA-responsive genes. Expression levels of *RD29A*, *RD20*, and *AHG3* were analyzed in WT and *gpl-1* mutant plants. Seven-day-old seedlings were treated with or without 50 μM ABA on MS medium at room temperature for 4 h. Total RNA was extracted, and RT–qPCR analysis was performed. *UBQ5* served as the internal control. Error bars represent SE. Statistical significance was determined by Student’s *t-*test (∗*p* < 0.05, ∗∗*p* < 0.01).

The ABA hypersensitivity and enhanced drought resilience of *gpl* mutants (Figures 2A–2D), together with the interaction of GPL with HOS15 and HDA6—both known regulators of histone modification (Park et al., 2018a; Figure 3D and Supplemental Table 2)—led us to hypothesize that GPL modulates ABA-responsive gene expression through epigenetic mechanisms, specifically histone modification. To test this, we conducted global RNA-seq analysis of WT and *gpl-1* mutant plants, with and without ABA treatment (Supplemental Dataset 1). RNA-seq analysis revealed that ABA-responsive genes were significantly upregulated in *gpl-1* mutants compared with WT following ABA treatment (Figure 4A and Supplemental Figure 10). In ABA-treated WT plants, 3,003 differentially expressed genes (DEGs) were identified, including 986 (32.8%) upregulated and 2,017 (67.2%) downregulated genes (Figure 4B). In contrast, 6,069 DEGs were identified in ABA-treated *gpl-1* plants, including 3,617 (59.6%) upregulated and 2,452 (40.4%) downregulated genes (Figure 4B). Notably, key ABA-responsive genes—including *ABI1*, *ABI2*, and *AHG3* (a protein phosphatase gene); *ABFs* and *ABI5* (transcription factors); and stress response marker genes *RD20*, *RD29A*, and *DREB2A*—were strongly induced in the *gpl-1* mutant compared with WT (Figures 4C and 4D; Supplemental Figure 11; Supplemental Dataset 1). Together, these results indicate that GPL functions as a negative regulator of ABA-responsive gene expression.

### GPL regulates histone H3K9 modification at the promoters of ABA-responsive genes

**Figure 5:**
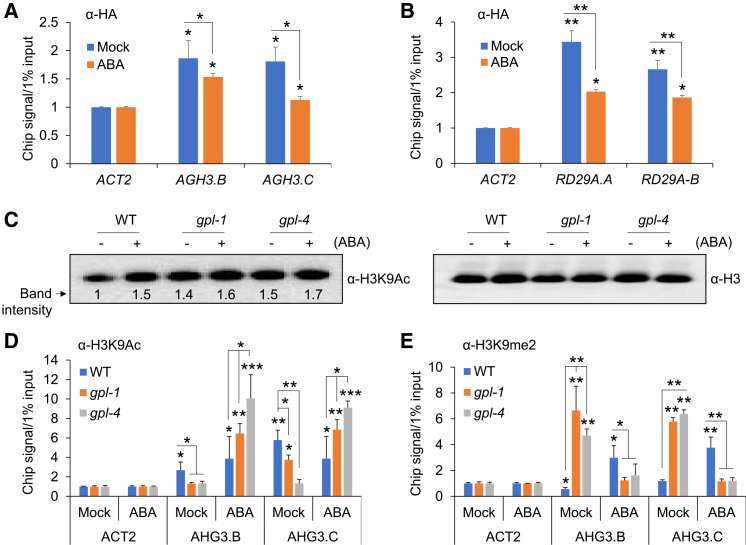
GPL associates with and negatively regulates the expression of ABA-responsive genes. **(A and B)** GPL associates with the *AHG3* and *RD29A* loci. Association of GPL with the target loci is reduced upon exposure to exogenous ABA (50 μM). The promoter regions of *AHG3***(A)** and *RD29A***(B)** were analyzed by ChIP–qPCR using anti-HA antibodies. *ACTIN2* served as the internal control. Error bars represent SE. Statistical significance was determined by Student’s *t-*test (∗*p* < 0.05, ∗∗*p* < 0.01). **(C)** GPL negatively regulates H3K9 acetylation, and *GPL* mutation leads to H3K9 hyperacetylation. Ten-day-old seedlings of WT (Col-0) and *gpl* mutants were treated with 50 μM ABA for 4 h. Histone H3 acetylation status was examined using anti-H3K9Ac antibodies. Histone H3 (H3) was used as a loading control. **(D)** H3K9 acetylation at the *AHG3* promoter is increased in *gpl* mutants upon ABA (50 μM) treatment. The promoter region of *AHG3* was analyzed by ChIP–qPCR using anti-H3K9Ac antibodies. ACTIN2 served as the internal control. Error bars represent SE. Statistical significance was determined by Student’s *t-*test (∗*p* < 0.05, ∗∗*p* < 0.01, ∗∗∗*p* < 0.005). **(E)** H3K9 dimethylation at the *AHG3* promoter is reduced in *gpl* mutants upon ABA treatment. The promoter region of *AHG3* was analyzed by ChIP–qPCR using anti-H3K9me2 antibodies. ACTIN2 served as the internal control. Error bars represent SE. Statistical significance was determined by Student’s *t-*test (∗*p* < 0.05, ∗∗*p* < 0.01).

In parallel with the RNA-seq analysis, we next asked whether GPL directly associates with the promoters of key ABA-responsive genes. To test this, a ChIP assay was carried out using anti-HA antibodies in *35S*::*GPL-HA* transgenic plants. GPL exhibited strong enrichment at the *AHG3* locus, which encodes a key ABA-responsive PP2C phosphatase, and at the *RD29A* locus, an ABA- and stress-responsive marker gene (Supplemental Figure 12A). In contrast, no significant enrichment was detected at the promoters of other ABA-responsive genes, including those encoding ABA phosphatases (*ABI1* and *ABI2*) and ABA-responsive transcription factors (*ABF1*, *ABF2*, *ABF4*, and *ABI5*), except for weak association with the *ABF3* promoter (Supplemental Figure 12B). Notably, ABA treatment significantly reduced GPL occupancy at the *AHG3* and *RD29A* loci (Figures 5A and 5B), suggesting that ABA signaling disrupts GPL binding to these loci, thereby alleviating transcriptional repression. Previous studies have shown that HOS15, in complex with PWR and HDA9, represses gene expression by promoting histone H3K9 deacetylation and methylation (Mayer et al., 2019; Zareen et al., 2022; Lim et al., 2023). To assess whether GPL contributes to this epigenetic regulation, we analyzed H3K9 acetylation levels in *gpl* mutants. Similar to *hos15-2*, *gpl* mutants exhibited hyperacetylation of H3K9 compared with WT (Figure 5C), suggesting that GPL helps maintain H3K9 in a deacetylated chromatin state and repress its transcription.

As HOS15, PWR, and HDA9 regulate gene expression through histone H3K9 deacetylation and methylation (Mayer et al., 2019; Zareen et al., 2022; Lim et al., 2023), we hypothesized that GPL similarly influences histone modifications at the promoters of ABA-responsive genes. To test this, we performed ChIP–qPCR using antibodies against H3K9 acetylation (H3K9Ac) and dimethylation (H3K9me2) to examine the epigenetic status of the *AHG3* locus in *gpl* and WT plants. Upon treatment with exogenous ABA, *gpl* mutants exhibited increased H3K9 acetylation and reduced H3K9 dimethylation at the *AHG3* locus compared with WT (Figures 5D and 5E). Similarly, H3K9 dimethylation was reduced at the stress-responsive *RD20* and *RD29A* loci in *gpl* mutants upon ABA exposure (Supplemental Figure 13). These results indicate that GPL promotes H3K9 dimethylation to repress specific ABA-responsive genes, whereas ABA-induced dissociation of GPL shifts the chromatin state toward H3K9 hyperacetylation, thereby facilitating transcriptional activation. Together, these findings demonstrate that GPL, as a component of the HOS15–PWR–HDA9/HDA6 complex, represses ABA-responsive gene expression by mediating targeted histone H3K9 modifications at specific promoters.

## Discussion

**Figure 6:**
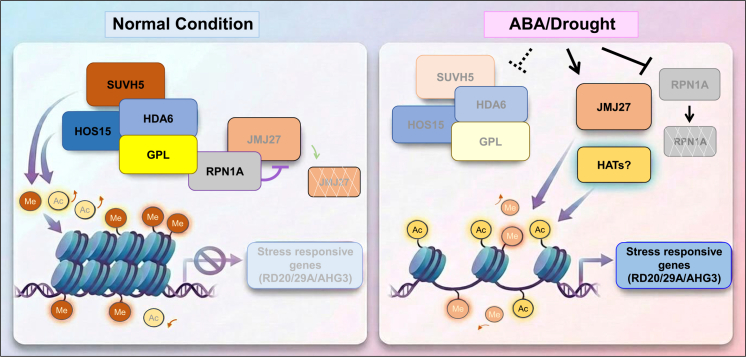
Hypothetical model illustrating the role of the GPL–HOS15 repressor complex. Under normal conditions, GPL forms a complex with HOS15, HDA6, and RPN1A, which negatively regulates the chromatin of stress-responsive genes through H3K9 deacetylation. At the same time, this complex also promotes H3K9 dimethylation (H3K9me2) at the same loci, reinforcing transcriptional repression. Upon drought or ABA treatment, GPL is rapidly destabilized, impairing its interaction with HOS15. RPN1A is also destabilized under drought stress, whereas JMJ27 is highly stabilized under stress conditions (Wang et al., 2021), demethylating H3K9 and promoting H3K9 acetylation, thereby activating the transcription of stress-responsive genes.

The GPL–HOS15–PWR–HDA9 complex is homologous to the animal NR–corepressor complex, which regulates key physiological processes by controlling target gene expression (Suzuki et al., 2018; Mayer et al., 2019; Zareen et al., 2022; Lim et al., 2023; Ali et al., 2025). In animals, the core component GPS2 plays key roles in transcriptional reprogramming across diverse cellular processes (Peng et al., 2001; Zhang et al., 2002; Lee et al., 2006; Cheng and Kao, 2009; Huang et al., 2015). Here, we identify GPL as the plant homolog of GPS2 and show that it negatively regulates plant development and stress responses (Figure 2 and Supplemental Figures 5D–5I). GPL forms a multiprotein complex through interactions with HOS15, HDA6, and RPN1A, a 26S proteasome regulatory subunit that negatively regulates drought stress via JMJ27 degradation (Figure 1, Figure 2, Figure 3, Figure 4, Figure 5, Figure 6 and Supplemental Table 2; Wang et al., 2021). The GPL–HOS15–HDA6 complex functions as an epigenetic rheostat that fine-tunes ABA and drought responses through localized histone modifications at stress-responsive loci. Under basal conditions, it promotes H3K9 deacetylation (via histone deacetylase activity) and maintains moderate levels of H3K9me2 at the promoters of key ABA/drought-responsive genes such as AHG3, RD29A, and RD20 (Figures 5D and 5E). Loss of GPL destabilizes the complex (Figures 3E and 3F), resulting in diminished nuclear accumulation of HOS15 and reduced promoter occupancy, even when protein levels are restored by proteasome inhibition (Supplemental Figure 9). This observation aligns with the constitutive derepression and ABA hypersensitivity observed in *gpl* mutants (Figures 2 and 4). Intriguingly, *gpl* mutants exhibit increased H3K9me2 and decreased H3K9 acetylation at these loci in the absence of ABA (Figures 5C–5E), a pattern that appears counterintuitive if GPL directly promotes H3K9me2 deposition. This likely reflects indirect compensatory effects or impaired recruitment of the repressive complex, allowing basal accumulation of repressive marks, while global hyperacetylation elsewhere (Figure 5C) contributes to the overall repression of the ABA transcriptional network (Figure 4).

Given that ABA treatment rapidly destabilizes the GPL–HOS15–HDA6 complex (Figures 3C and 3D), this is expected to favor derepression by reducing histone deacetylation and limiting the maintenance of H3K9me2. However, we observed a modest increase in H3K9me2 at the *AHG3* locus in ABA-treated WT plants (Figure 5E), alongside the expected locus-specific hyperacetylation in *gpl* mutants (Figure 5D). This apparent discrepancy can be explained by stress-induced activation of the H3K9 demethylase JMJ27, which is negatively regulated by RPN1A under normal conditions (Wang et al., 2021). ABA and drought destabilize RPN1A, thereby stabilizing JMJ27 and promoting active H3K9 demethylation at target loci (including RD20 and related genes). This process ultimately overrides residual methylation and drives robust transcriptional activation (Wang et al., 2021). In *gpl* mutants, the absence of the repressive complex elevates basal gene expression, whereas the JMJ27-dependent demethylation pathway remains functional, resulting in amplified transcriptional responses under stress conditions.

Importantly, our conclusions pertain to locus-specific, low-amplitude changes detected by targeted ChIP–qPCR at euchromatic stress-responsive promoters, rather than genome-wide patterns in which H3K9me2 predominantly marks heterochromatin and transposable elements, with minimal enrichment at RD29A, RD20, or AHG3 in public ChIP-sequencing (ChIP-seq) datasets. Such localized and dynamic regulation of H3K9me2 at specific ABA/drought loci is supported by prior evidence that JMJ27-mediated demethylation promotes a permissive chromatin state under stress (Wang et al., 2021). Thus, the GPL–HOS15 complex does not impose broad euchromatic silencing but instead functions as a context-dependent modulator: under normal conditions, it maintains repression via deacetylation and limited methylation, whereas ABA shifts the balance toward activation through complex disassembly and JMJ27 activity (Figure 5F). This multilayered mechanism allows precise, rapid, and reversible control of stress-responsive transcription, highlighting GPL as a key integrator of plant drought adaptation.

Notably, GPL stabilizes HOS15 and facilitates its transcriptional regulatory activity, demonstrating their interdependent stability (Figure 3 and Supplemental Figure 9). ABA exposure destabilizes the GPL–HOS15 complex, thereby relieving repression and permitting rapid activation of stress responses. Our results complement previous studies of HOS15, which functions both as an E3 ligase adaptor and as a chromatin regulator (Park et al., 2018a; Shen et al., 2020; Lim et al., 2021). Unlike HOS15, GPL lacks ubiquitin E3 ligase activity and instead specializes in maintaining chromatin repression under non-stress conditions. Together, GPL and HOS15 constitute a versatile module linking histone modification with stress signaling. The discovery of GPL as the plant homolog of animal GPS2 completes the conserved repressor complex in plants, filling a long-standing gap. This finding supports the concept that chromatin-based repression mechanisms are evolutionarily conserved yet adapted to lineage-specific challenges, such as drought adaptation in sessile plants (Figure 6).

Previous studies have shown that PWR and HDA9 form a complex with ABI4 that promotes ABA and drought responses by repressing ABA catabolic genes such as *CYP707A*s (Khan et al., 2020). In contrast, HOS15 represses ABA and drought responses by modulating stability of the OST1 kinase and the DIL9 transcription factor (Ali et al., 2019; Zareen et al., 2025). Although GPL interacts with, and likely functions within, the same complex as PWR, HDA9, and HOS15 during developmental processes (Supplemental Figures 4D–4I), it exhibits *hos15-2*-like phenotypes, including ABA hypersensitivity and enhanced drought tolerance (Figure 2; Ali et al., 2019). By contrast, *hda9* and *pwr* mutants display ABA insensitivity and drought sensitivity (Khan et al., 2020). We propose that PWR and HDA9 primarily act upstream to repress ABA catabolic genes (such as *CYP707A*s), thereby maintaining elevated ABA levels and contributing to ABA insensitivity in their respective mutants (Baek et al., 2020; Khan et al., 2020). In contrast, GPL and HOS15 directly repress ABA-responsive output genes, including *RD29A*, *RD20*, and *AHG3*, leading to enhanced ABA responses in *gpl* and *hos15* mutants. This framework reconciles the observed phenotypic differences and supports a context-dependent division of labor within the shared corepressor complex. Together, these observations indicate that the HOS15–GPL and PWR–HDA9 complexes constitute two distinct functional modules with opposing roles in ABA and drought responses. This distinction likely reflects a multilayered organization of the corepressor complex and the hierarchical regulation of stress-response pathways involving PWR, HDA9, HOS15, and GPL.

Beyond its evolutionary significance, GPL also has potential applications. The enhanced drought resilience of *gpl* mutants suggests that modulating GPL activity could improve crop stress tolerance. However, the dwarfism and developmental defects observed in these mutants underscore GPL’s dual role in coordinating growth and stress responses. Future studies employing genome-wide ChIP-seq analyses and identifying E3 ligases that regulate GPL will be critical to define its targets and enable strategies for crop improvement without compromising development. In conclusion, GPL functions as a central chromatin regulator within the HOS15 repressor complex, dynamically balancing growth, ABA signaling, and drought resilience. This work establishes the long-missing link between plant and animal chromatin repression complexes and opens new opportunities for epigenetic approaches to climate-resilient agriculture.

## Methods

### Plant materials and growth conditions

*Arabidopsis thaliana* ecotype Col-0 was used as the WT in this study. The *hos15-2* and *hda6* (*rts1-1*/*axe1-5*) alleles have been described previously (Murfett et al., 2001; Aufsatz, 2002; Park et al., 2018a). For growth assays, seeds were surface-sterilized and germinated on half-strength Murashige and Skoog (½ MS) medium supplemented with 2% sucrose and solidified with either 0.25% Phytagel (for horizontal plates) or 1.2% agar (for vertical plates). Plants were grown at 23°C under long-day conditions in a controlled culture room.

### Vector construction and generation of transgenic plants

To generate *GPL-OX* transgenic plants, the coding sequence of *GPL* was cloned into the *pDONR*/*Zeo* Gateway entry vector (Invitrogen, Carlsbad, CA, USA) and subsequently recombined into the destination vector pGWB14 (Invitrogen). The resulting constructs were introduced into WT or *hos15-2* plants via *Agrobacterium tumefaciens-*mediated floral dipping. Primers used for cloning are listed in Supplemental Table 3.

### Generation of *GPL*-CRISPR lines

*GPL-*CRISPR lines were generated as previously described (Liu et al., 2015). Sequences of GPL single-guide RNAs (sgRNAs) are listed in Supplemental Table 3. To confirm stable mutants, we carried out three different assays: (1) DNA sequencing analysis, (2) amplified fragment length polymorphism analysis, and (3) genotyping (for detailed protocols, see Liu et al., 2015). Multiple independent transgenic lines harboring CRISPR–Cas9 constructs targeting *GPL exhibited* a deletion of approximately 900 bp between the two sgRNA target sites. Two representative lines (*gpl-1* and *gpl-4*) were used for experiments.

### Germination and root growth assessment

For phenotypic analysis under ABA treatment, seeds of WT and the indicated genotypes were surface-sterilized and germinated on ½ MS medium supplemented with the indicated concentrations of ABA (μM). Plates were stratified at 4°C for 2 days and then transferred to a chamber at 22°C. For the root growth assay, seeds were plated on ½ MS medium (vertical plates), and after 4 days, seedlings were transferred to new plates containing ABA or NaCl. Photographs were taken after 7 days.

### Drought treatment

Drought-stress assays were performed as previously described (Ali et al., 2019). Briefly, seeds of WT and the selected genotypes were surface-sterilized, germinated on ½ MS medium for 1 week, and transferred to soil. Drought tolerance was assessed in 3-week-old plants by withholding water for 14 days, followed by rewatering. Photographs were taken 3 days after rewatering. Survival rates were determined after drought stress.

### Measurement of stomatal aperture

Two-week-old seedlings of the indicated genotypes were floated on stomatal opening solution (2-(*N-morpholino)ethanesulfonic acid* + KCl buffer) at room temperature for 1 h, as described by Li et al. (2013). Half of the samples were then treated with 10 μM ABA (+ABA), while the remaining half served as controls (–ABA) and were further incubated for 2 h. At least 10 stomata from each genotype were observed using a 40× objective lens and a Leica ICC50 E microscope camera. These experiments were repeated twice with similar results.

### RNA isolation and RT–qPCR analysis

Total RNA was extracted from seedlings using the RNeasy Plant Mini Kit (Qiagen, Germantown, MD, USA) and treated with DNase (Sigma, St. Louis, MO, USA). First-strand cDNA was synthesized using the Thermoscript RT–PCR system (Invitrogen, Paisley, UK). PCR amplification was performed using e-Taq DNA polymerase (Solgent, Daejeon, Korea). Primers used for RT–PCR and real-time PCR are listed in Supplemental Table 3. The conditions of real-time PCR were as follows: 95°C for 5 min; 45 cycles of 95°C for 10 s and 60°C for 30 s; followed by 95°C for 10 s, 65°C for 5 s, and 95°C for 5 s.

### RNA-seq analysis

For RNA-seq analysis, approximately 40 whole 14-day-old seedlings from each genotype and treatment were collected and pooled as a single sample for RNA extraction. Library construction and deep sequencing were performed using the Illumina HiSeq 3000 platform (Theragen Bio, Seongnam, Korea). Paired-end RNA-seq reads were aligned to the *A. thaliana* genome (TAIR10) using Bowtie2 (Langmead and Salzberg, 2012). Gene transcript abundance was quantified using featureCounts (Liao et al., 2014). Fold change and false discovery rate (FDR) were calculated using DESeq2 (Love et al., 2014). DEGs at each time point compared with 0 h were identified using the following criteria: log_2_(fold change) > 1 and FDR < 0.05 for upregulated genes, and log_2_(fold change) < −1 and FDR < 0.05 for downregulated genes. Gene Ontology analysis was performed using the AgriGO and PGSEA tools (Kim and Volsky, 2005). The Degust site (https://degust.erc.monash.edu/) (Powell, 2019) was used to analyze the expression of ABA-related genes (Supplemental Figure 11).

### ChIP assay

ChIP assays were performed as previously described (Park et al., 2018a). Briefly, 14-day-old seedlings were crosslinked with 4% formaldehyde for 10 min under vacuum. The crosslinked samples were then neutralized with 0.1 M glycine and washed three times with dH_2_O. Seedlings were ground in liquid N_2_ and resuspended in modified RIPA buffer. The DNA was fragmented to ∼500–1000 bp by sonication. The DNA fragments were precleared with salmon sperm DNA/protein A agarose for 60 min at 4°C, followed by immunoprecipitation with α-HOS15 (endogenous α-HOS15 serum), α-HA (Merck Roche, #11867431001), α-H3K9Ac (Agrisera, #AS16 3198), or α-H3K9me2 (Abcam, #ab1220) antibodies. Immunocomplexes were washed and eluted using elution buffer. Crosslinks were reversed by incubation at 65°C for 6 h, followed by degradation with protease K to remove residual proteins. DNA was extracted with phenol/chloroform/isoamyl alcohol, precipitated with ethanol, and resuspended in Tris–EDTA buffer. For qPCR analysis, 2 μl of the precipitated DNA was used to determine the amount of genomic DNA in ChIP experiments.

### Affinity purification via IP–MS

Total proteins were extracted from 5 g of 2-week-old seedlings as described previously (Park et al., 2018b). α-HA magnetic beads (M18011; MBL International, Woburn, MA, USA) were used to immunoprecipitate GPL-HA and its interacting proteins, which were then analyzed by liquid chromatography–mass spectrometry.

### Immunoblot analysis and co-IP

Ten-day-old *Arabidopsis* plants, either treated with ABA/CHX/MG132 or untreated, were used for western blot analysis. Proteins were extracted, and immunoblotting was carried out using α-HOS15, rat α-HA, α-HDA6 (Phyto AB, #PHY2875A), α-RPN1A (Phyto AB, #PHY2594A), α-H3 (Abcam, #ab1791), and α-H3K9Ac antibodies. All antibodies are commercially available. For co-IP, HA-tagged 35S::*GPL Arabidopsis* plants were used. Total proteins were extracted and immunoprecipitated with α-HA, followed by immunoblotting with α-HA, α-HOS15, α-HDA6, or α-RPN1A. Each immunoblot was incubated with the appropriate primary antibody for 2 h at room temperature or overnight at 4°C. Membranes were developed using peroxidase-conjugated secondary antibodies at 1:1000 for anti-rat immunoglobulin G (IgG) (Santa Cruz Biotechnology, Santa Cruz, CA, USA) and 1:3000 for anti-rabbit antibodies (GE, Little Chalfont, UK). For immunoprecipitation in tobacco transient assays, leaves were co-infiltrated with HA-tagged GPL and GFP-tagged HOS15. After 3 days, total protein was extracted and pulled down with α-GFP (Merck Sigma-Aldrich, #SAB4301138), followed by immunoblotting with α-HA or α-GFP (Abcam, #ab13970). Membranes were incubated with the appropriate primary antibodies (α-HA, 1:2000; α-GFP, 1:5000) for 2 h at room temperature or overnight at 4°C, and developed using peroxidase-conjugated secondary antibodies at 1:1000 for anti-rat IgG (Sigma) and 1:4000 for anti-rabbit antibodies (GE).

### Nuclear cytoplasmic fractionation assay

Nuclei were isolated from 2-week-old plants using Honda buffer composed of 0.4 M sucrose, 2.5% Ficoll 400 (Sigma), 5% dextran T-40 (Sigma), 10 mM MgCl_2_, 25 mM Tris–Cl (pH 7.5), 10 mM β-mercaptoethanol, 100 mg/ml phenylmethylsulfonyl fluoride, 0.5 mg/ml antipain, and 0.5 mg/ml leupeptin. Following filtration through a 60-μm nylon mesh (Millipore), samples were chilled on ice for 15 min. Triton X-100 was then added to a final concentration of 0.5%, and the mixture was centrifuged at 1500 × *g* for 5 min. The resulting supernatant, containing cytosolic proteins, was collected. The remaining pellet was washed with Honda buffer containing 0.1% Triton X-100, gently resuspended, and centrifuged at 100 × *g* for 5 min to remove starch and debris. The supernatant was subsequently centrifuged at 1800 × *g* for 5 min to collect nuclei. Immunoblotting was carried out using α-HOS15, α-tubulin (Sigma), and α-H3 antibodies, and protein detection was performed using an imaging system (ChemiDoc MP; Bio-Rad, Hercules, CA, USA).

### Y2H assay

The full-length *GPL* coding sequence was cloned into the Gateway entry vector *pDONR*/*Zeo* and subsequently recombined into the Y2H bait (BD) vector pDEST32. PWR was similarly cloned into *pDONR*/*Zeo* and recombined into the prey (AD) vector pDEST22. Primers used for cloning are listed in Supplemental Table 3. The resulting constructs were transformed into the yeast strain PJ694A. Three independent transformants carrying the *GPL* construct were tested for interaction with PWR. Empty vectors were used as negative controls.

## Data and code availability

Raw RNA-seq data generated in this study have been deposited in the NCBI Gene Expression Omnibus (GEO) under accession numberGSE326317.

## Funding

This work was supported by grants from the 10.13039/501100003725National Research Foundation of Korea funded by the Ministry of Science and ICT (MSIT) (RS-2024-00407469 to D.-J.Y. and J.P.; RS-2022-NR070541 to D.-J.Y.; RS-2025-23963699 to A.A.; and RS-2023-00239735 to J.P.), as well as by the 10.13039/501100003336Bulgarian National Science Fund (project CAFTA, grant no. КП06 ДВ/2 ЦС to A.A.).

## Acknowledgments

We thank Prof. Byeong-Ha Lee and Dr. Si-in Yu (Sogang University, Korea) for providing the CRISPR–Cas9 vector and assistance in generating *GPL*-CRISPR lines. Figure 6 was created using the online tool Figurelabs (https://chat.figurelabs.ai/chat). No conflict of interest declared.

## Author contributions

A.A. and D.-.J.Y. designed the research; D.-J.Y. supervised the study; A.A., S.Z., K.P., I.U.K., M.J.B., and Z.-Y.X. performed the experiments; Z.E.B., N.A., and J.P. analyzed RNA-seq data; A.A., Z.-Y.X., and D.-J.Y. analyzed the data and wrote the manuscript; and R.A.B. and J.M.P. edited the manuscript. All authors reviewed and approved the final manuscript.

## Supplemental information

Document S1. Supplemental Figures 1–14 and Supplemental Tables 1–3

Supplemental Dataset 1. RNA-seq dataset S1

Document S2. Article plus supplemental information
