# Supplementary material for: A GPS2-like protein interacts with HOS15 and HDA6 to form a repressor complex that regulates ABA signaling and drought adaptation in Arabidopsis
Source: Plant Commun. 2026 Apr 3;7(6):101843. doi: 10.1016/j.xplc.2026.101843 (PMC13261656; doi:10.1016/j.xplc.2026.101843)
Supplement: Document S1. Supplemental Figures 1–14 and Supplemental Tables 1–3 [file mmc1.pdf]

Plant Communications, Volume 7

Supplemental information

A GPS2-like protein interacts with HOS15 and HDA6 to form a repressor complex that regulates ABA signaling and drought adaptation in *Arabidopsis*

Akhtar Ali, Shah Zareen, Zein Eddin Bader, Junghoon Park, Irfan Ullah Khan, Kisuk Park, Nassem Albakri, Min Jae Bae, Ray A. Bressan, Jose M. Pardo, Dae-Jin Yun, and Zheng-Yi Xu

Supplemental information

A GPS2-like protein interacts with HOS15 and HDA6 to form a repressor complex that regulates ABA signaling and drought adaptation in Arabidopsis

Akhtar Ali[1,2,7,*], Shah Zareen[1,7], Zein Eddin Bader[1], Junghoon Park[1], Irfan Ullah Khan[3], Kisuk Park[1], Nassem Albakri[1], Min Jae Bae[1], Ray A. Bressan[4], Jose M. Pardo[5], Dae-Jin Yun[1,*], Zheng-Yi Xu[6,*]

[1] School of Advanced Biotechnology, Plant Global Stress Research Center, Konkuk University, Seoul 05029, South Korea. [2] Department of Molecular Stress Physiology, Center of Plant Systems Biology and Biotechnology, Plovdiv, Bulgaria. [3] School of Biological Sciences, Seoul National University, Seoul 08826, Korea. [4] Department of Horticulture and Landscape Architecture, Purdue University, 625 Agriculture Mall Dr., West Lafayette, IN 47907-2010 USA. [5] Instituto de Bioquimica Vegetal y Fotosintesis, cicCartuja, CSIC-Universidad de Sevilla, Americo Vespucio 49, Sevilla, 41092, Spain. [6] Key Laboratory of Molecular Epigenetics of the Ministry of Education (MOE), Northeast Normal University, Changchun, China. [7]These authors equally contributed to this work.

*For Correspondence;

Akhtar Ali aali@konkuk.ac.kr, Dae-Jin Yun djyun@konkuk.ac.kr, Zheng-Yi Xu xuzy100@nenu.edu.cn,

Supplementary information

Supplementary Fig. 1. GPL shows protein sequence similarities with animal GPS2.

Supplementary Fig. 2. Alphafold-3 protein structure of GPL and Human GPS2.

Supplementary Fig. 3. Phylogenetic analysis of GPL homologs in plants.

Supplementary Fig. 4. Protein interaction network of GPL using IP-MS.

Supplementary Fig. 5. Generation of CRISPR/Cas9-mediated *gpl*-mutant lines.

Supplementary Fig. 6. GPL negatively regulates ABA response.

Supplementary Fig. 7. *GPL*-overexpression lines show ABA insensitive phenotypes.

Supplementary Fig. 8. GPL stability is dependent on functional HOS15.

Supplementary Fig. 9. Functional GPL is required for HOS15 stability and activity.

Supplementary Fig. 10. GO terms analysis of GPL-regulated genes (Clusters A-F).

Supplementary Fig. 11. ABA related genes are up-regulated in *gpl-1*, (RNA-seq analysis).

Supplementary Fig. 12. Association of GPL with the promoters of ABA responsive genes.

Supplementary Fig. 13. GPL regulates H3K9me2 at the promoters of ABA responsive genes.

Supplementary Fig. 14. GPL interacts with RPN1A in Arabidopsis.

Supplementary Table 1. GPL homolog from plant species.

Supplementary Table 2. GPL-interacting protein identified through IP-MS.

Supplementary Table 3. Primers sequences.

Supplementary Dataset 1. RNA-seq dataset S1 (separate file).

A

```
GPL           MQVEIPSPYDHGSRRQRRIRRTEETQKQKKMVAISMYRGNLHKVPDVPRRWIMPDRNLSF	60
GPS2          ------------------------------------------------------------	0


GPL           KDFKSLLHRRKKALSRLPLNPNLNLSVKTELVTDQENPILPSEANGSSGKQKLFEVKREE	120
GPS2          --MPALL----------------------------ERPKLSNAMARALHRHIMMERERK-	29
                : :**                            *.* * .    :  :: ::* :*:

GPL           ICGNRVKGDENNDRGFEGARSDGGDRPGRVTESKETDNVPHKYAAKEEE---TNEAAEKV	177
GPS2          -----------------------------RQEEEEVDKMMEQKMKEEQERRKKKEMEERM	60
                                             *.:*.*:: .:   :*:*   .:*  *::

GPL           -PSETELKRKEVEERLQVLNAKKHNLVQVLKQILNAEEELKRRSYMQQQGT-TVATRPSL	235
GPS2          SLEETKEQILKLEEKLLALQEEKHQLFLQLKKVLHEEEKRRRKEQSDLTTLTSAAYQQSL	120
                .**: :  ::**:* .*: :**:*.  **::*: **: :*:.  :     :.* : **

GPL           PLHVDVSN-DSGGNVGTHMEGGETDDAAN---HNNAQTRTLLRLCGASSSS-ESPLRRAA	290
GPS2          TVHTGTHLLSMQGSPGGHNRPGTLMAADRAKQMFGPQVLTTRHYVGSAAAFAGTPEHGQF	180
               :*...   .  *. * * . *    * .     . *. *  :  *::::   :* :

GPL           ALSQHNMVPHTSRWSPLVGPSQPGPAVTVS------ASGTNYIASSPSPAGFGGTSVFRE	344
GPS2          -QGSPGGAYGTAQPPPHYGPTQPAYSPSQQLRAPSAFPAVQYL-SQPQPQPYAVHGHFQP	238
                .. . .  *::  *  **:**. : : .        ..:*: *.*.*  :.  . *:

GPL           SR--L---------------------------QSPWN-----------------------	352
GPS2          TQTGFLQPGGALSLQKQMEHANQQTGFSDSSSLRPMHPQALHPAPGLLASPQLPVQMQPA	298
              ::  :                             * :

GPL           ------------------------------ 352
GPS2          GKSGFAATSQPGPRLPFIQHSQNPRFYHK 327
```

B

```
GPL           EEETNEAAE-KVPS-----------------ETELKRKEVEERLQVLNAKKHNLVQVLKQ	42
Hs            EEEVDKMMEQKMKEEQERRKKKEMEERMSLEETKEQILKLEEKLLALQEEKHQLFLQLKK	60
Mm            EEEVDKMMEQKMKEEQERRKKKEMEERMSLEETKEQILKLQEKLSALQEEKHQLFLQLKK	60
Dr            EEEVDKMMEQKLKEEEERKRKKEMEERMSLEETKEQILKLGVKLQGLQEEKHQLFLQLKK	60
              ***.::  * *: .                 **: :  ::  :*  *: :**:*.  **:

GPL           ILNAEEELKRRSYMQQQ-GTTVATRPSLPLH	72
Hs            VLHEEEKRRRKEQSDLTTLTSAAYQQSLTVH	91
Mm            VLHEEEKRRRKEQSDLTTLTSAAYQQSLTVH	91
Dr            VLHEEEKRRRKEQSDMTTLTSATYQANMPIH	91
              :*: **: :*:.  :    *:.: : .: :*
```

Figure S1. GPL shows protein sequence similarities with animal GPS2. (A) GPL shows 26.7% sequence similarity with human GPS2. Protein sequences of Arabidopsis GPL and human GPS2 were aligned using CLUSTAL OMEGA software. **(B)** GPL showed 59.75 sequence identity in the conserved motif with that of GPS2 from human (Hs; Homo sapiens), mouse (Mm; Mus musculus) and zebra fish (Dr; Danio rerio). Protein sequence of Arabidopsis GPL and human GPS2 were aligned using CLUSTAL OMEGA software (https://www.ebi.ac.uk/jdispatcher/msa/clustalo).

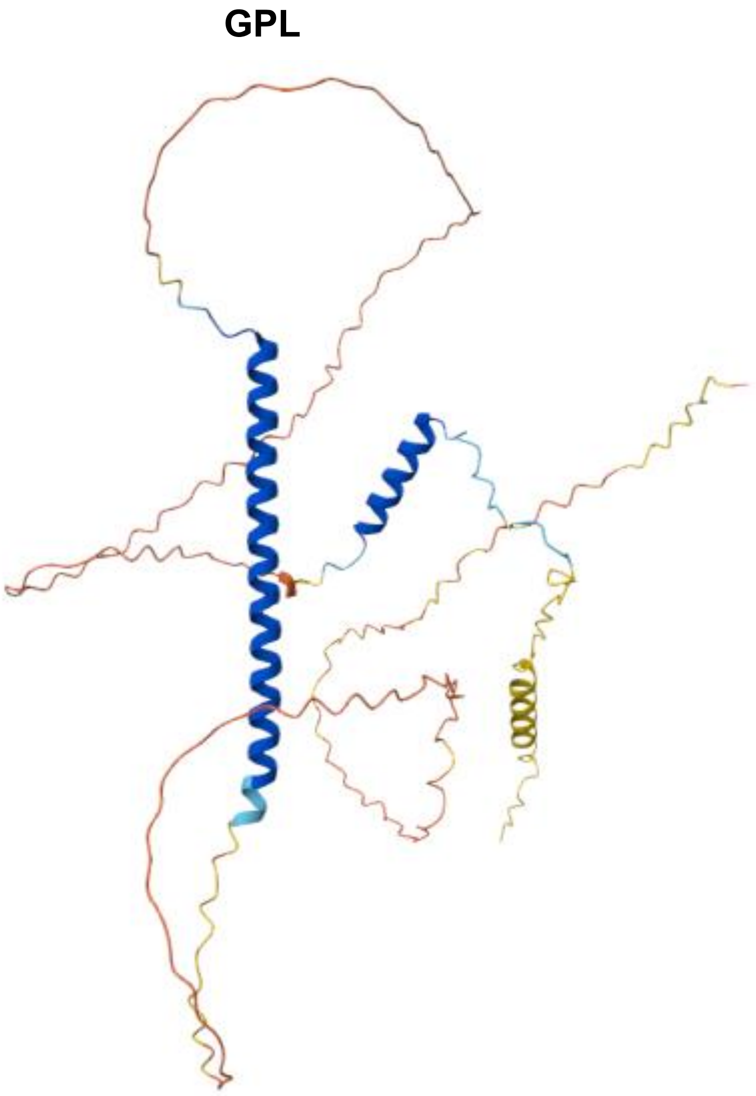

Figure S2. Alphafold-3 Protein structure of GPL and Human GPS2. GPL and GPS2 show comparable protein structure (Alphafold 3 https://alphafoldserver.com/).

A

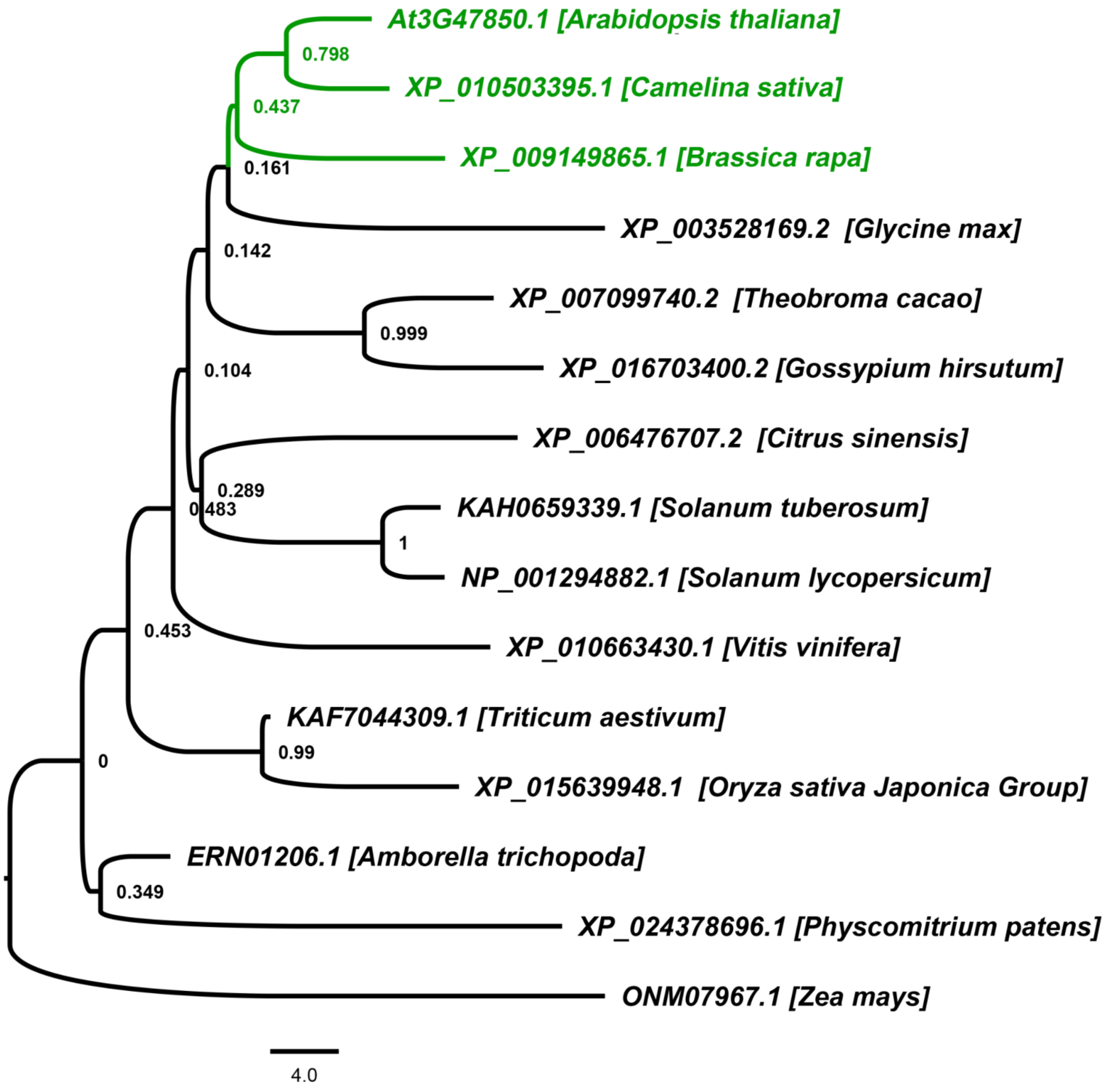

B

Plant species	GENE ID	Accession Length	% identities
Camelina sativa	XP_010503395.1	352	88.06%
Brassica rapa	XP_009149865.1	332	75.76%
Glycine max	XP_003528169.2	352	68.09%
Theobroma cacao	XP_007099740.2	346	64.41%
Gossypium hirsutum	XP_016703400.2	343	67.86%
Citrus sinensis	XP_006476707.2	364	63.64%
Solanum tuberosum	KAH0659339.1	326	70.91%
Solanum lycopersicum	NP_001294882.1	326	70.91%
Vitis vinifera	XP_010663430.1	361	62.86%
Triticum aestivum	KAF7044309.1	189	53.49%
Oriza sativa (Japonica)	XP_015639948.1	351	52.83%
Amborella trichopoda	ERN01206.1	166	55.81%
Physcomitrium patens	XP_024378696.1	708	47.22%
Zea mays	ONMO7967.1	219	54.76%

Figure S3. Phylogenetic analysis of GPL homologs in plants. Amino acid sequences of GPL homologs were aligned using ClustalW. The phylogenetic tree was generated using MEGA-11 software with the neighbor-joining method that is based on full-length proteins of GPL homologs from different plant species. The bar indicates the relative divergence of the sequences examined and bootstrap values from 1,000 replicates were displayed next to the branch. Brassicaceae species are shown in green. **(B)** Protein sequences percentage similarities of GPL with its homologs in different plant species as shown in "A".

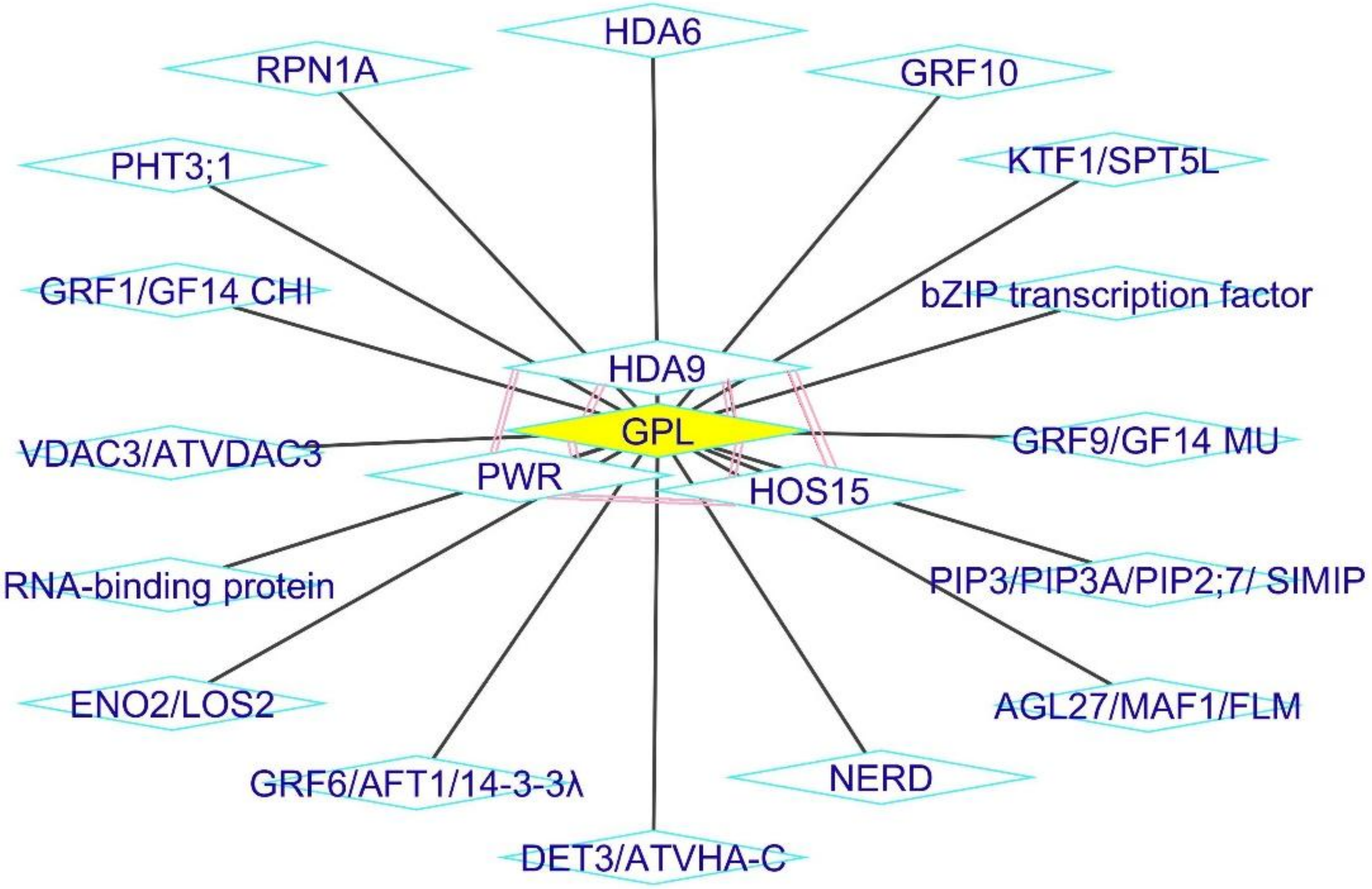

Figure S4. Protein interaction network of GPL using IP-MS. The GPL immunoprecipitation-mass spectrometry (IP-MS) interaction network was visualized using Cytoscape.js. The IP-MS analysis demonstrated that GPL forms a stable complex with the corepressor proteins PWR, HDA9, and HOS15. Beyond this core complex, GPL is also associated with numerous other proteins involved in various physiological processes and responses to environmental stress (for details see Supplementary Table 2).

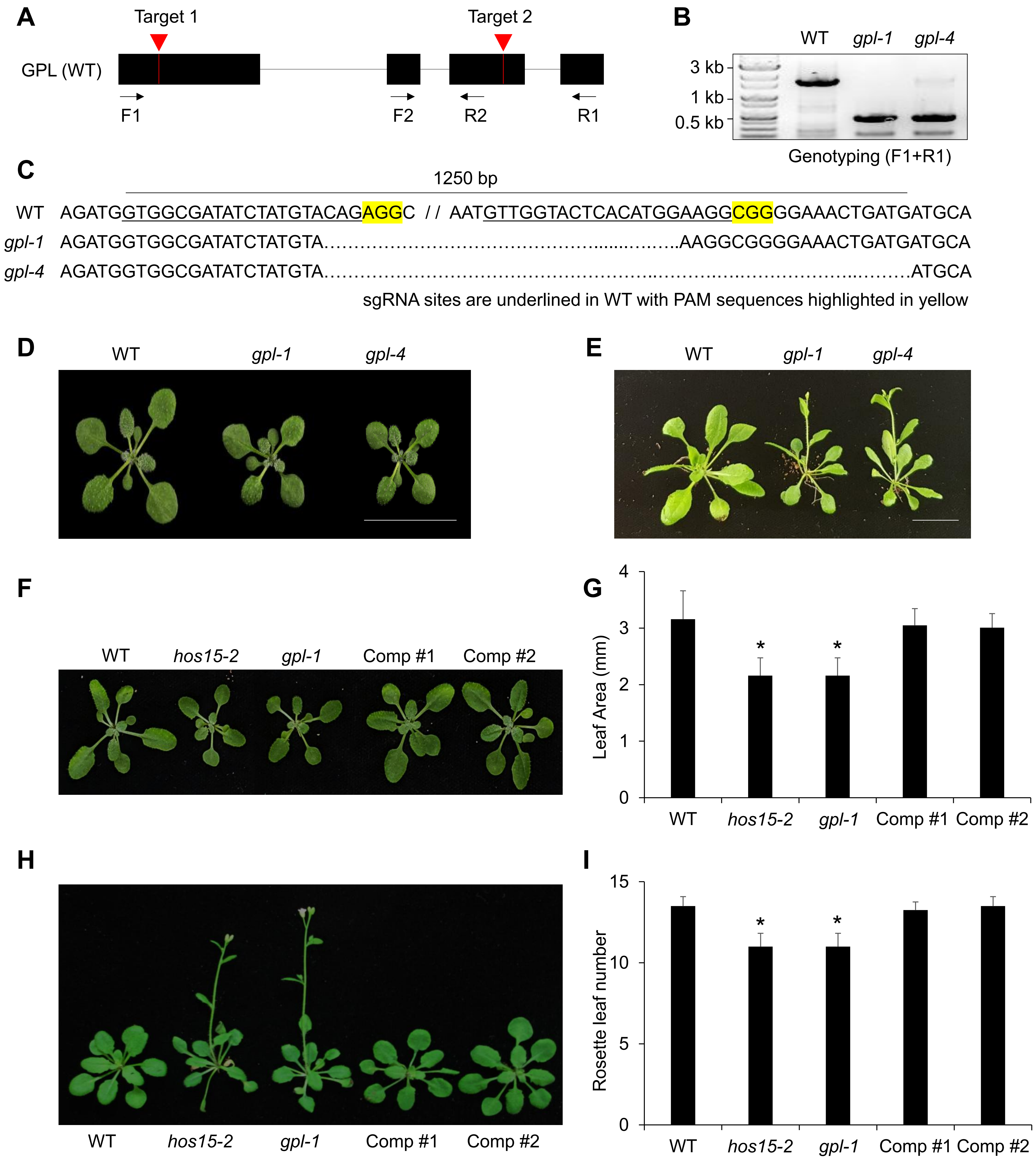

Figure S5. Generation of CRISPR/Cas9-mediated *gpl*-mutant lines. **(A)** Schematic diagram of GPL gene with two sgRNA positions as indicated by Target 1 and Target 2. **(B)** Genotyping of *gpl*-CRISPR lines for confirmation of *GPL*-mutation. After deletion of the middle region between two sgRNAs (as shown in A), the two sites bound together through NHEJ DNA repair system (non-homologous end joining) and as a result a small sized nonfunctional *GPL* gene was generated. **(C)** Sequence analysis of *gpl*-CRISPR lines for confirmation of *GPL*-mutation. In the two mutant lines more than 1200 bp region were deleted. sgRNA sites are underlined. **(D,E)** *gpl*-mutants show dwarf phenotypes **(D)**, and early flowering **(E),** compared to WT. **(F-I).** Dwarfism **(F,G)**, and early flowering **(H,I)** of *gpl*-mutant were rescued by expressing functional *GPL* in *gpl-1* mutant background under the control of *35S* promoter. *hos15-2* was used as experimental control.

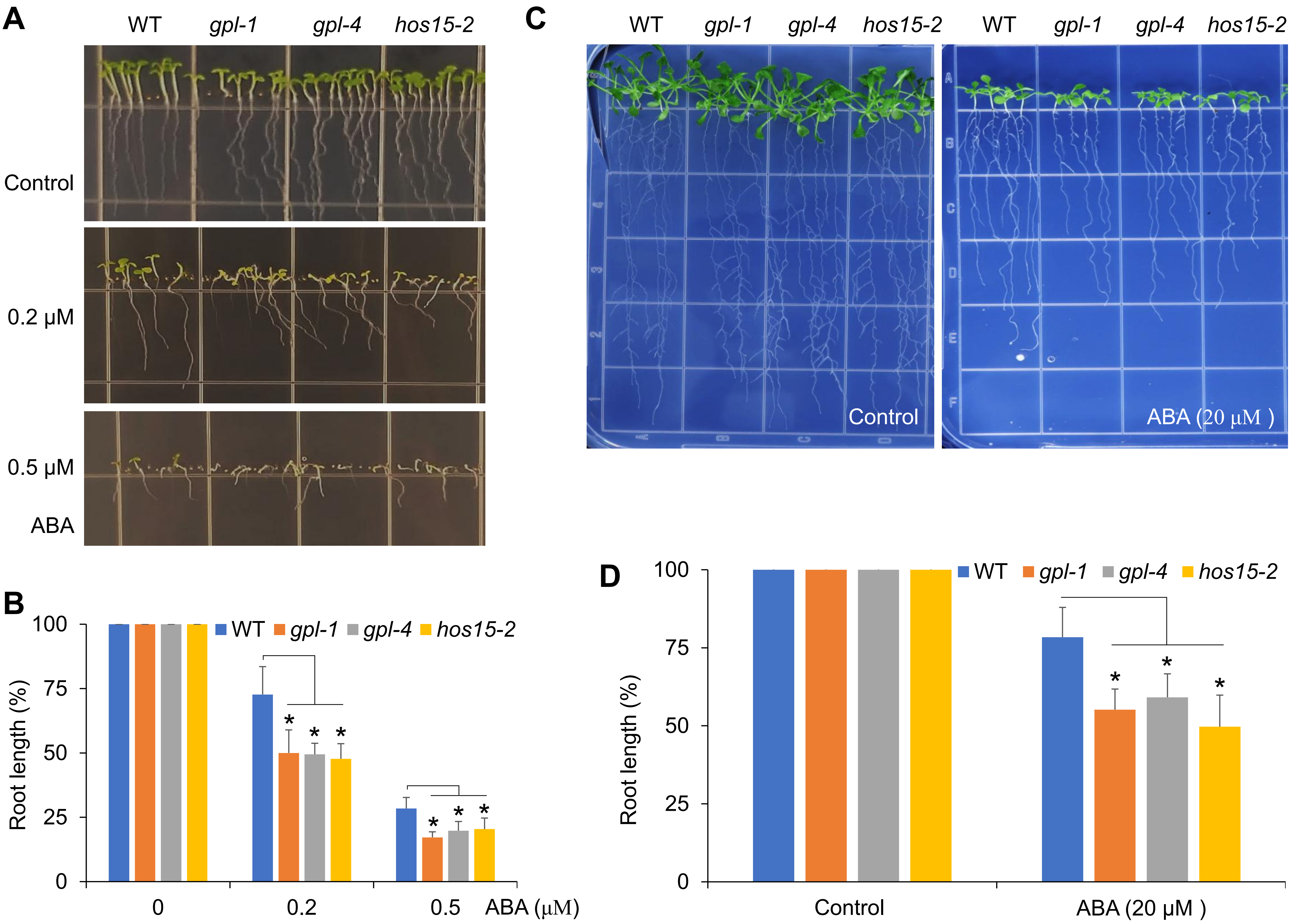

Figure S6. GPL negatively regulates ABA response. (A) *gpl*-mutation leads to ABA sensitivity. Seeds of indicated lines were germinated on MS media including ABA (μM). Photographs were taken after 6-days of germination. **(B)** Statistical analysis of root length in (A). Error bars represent SE. Significant difference was determined by Student's t-test with a P-value <0.05 (*). **(C)** 4-day old seedlings of indicated genotypes were transferred to control or ABA containing (20 μM) MS medium and allowed to grow for 1-week. Photographs were taken 7 days after transfer. **(D)** Statistical analysis of root length in (C). Error bars SE represent SE (B, D). Significant difference was determined by Student's t-test with a P-value <0.05 (*).

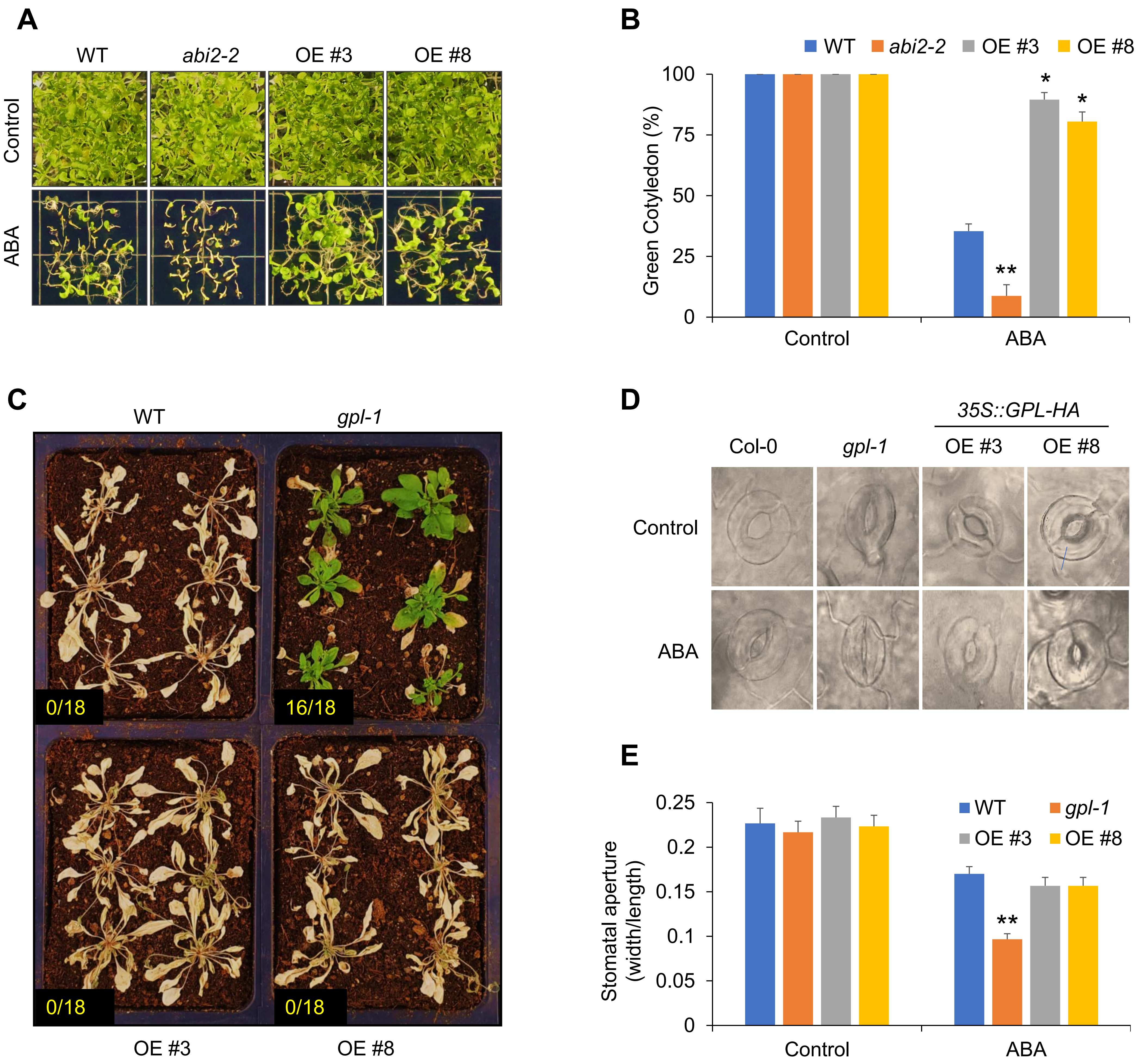

Figure S7. *GPL*-overexpression lines show ABA insensitive phenotypes. (A) *GPL*-overexpression leads to ABA insensitivity. Seeds of WT, transgenic lines overexpressing *35S::GPL-HA* (OE #3, OE #8) and *abi2-2* were germinated on 1/2 MS medium supplemented with ABA (0.75 μM). Photographs were taken 10-days after germination. *abi2-2* mutant was used as an experimental control. **(B)** Green cotyledons were counted after 10 days, with error bars representing SE (n=3). Significant difference was determined by Student's t-test with a P-value <0.05 (*). **(C)** *GPL*-overexpression lines (OE) show WT-like phenotypes under drought stress. Seeds of WT, *gpl*-1 and *GPL-OE* lines were germinated on 1/2 MS medium for 1-week and then transferred to soil. Drought tolerance assay of 3-week-old plants was performed by withholding water for 14 d and subsequently re-watered. Photographs were taken 3 days after re-watering. Survival rate of 3 pots of each of the indicated lines after drought test was measured. **(D)** *GPL-OE* lines show WT-like stomatal movement upon exposure to ABA (10 μM), whereas gpl-1 showed rapid stomatal closure upon exposure to ABA. **(E)** Stomatal size was measured using image-J software (width/length), with error bars representing SE (n=10 stomata per genotype. Significant difference was determined by student's t-test (**p < 0.01).

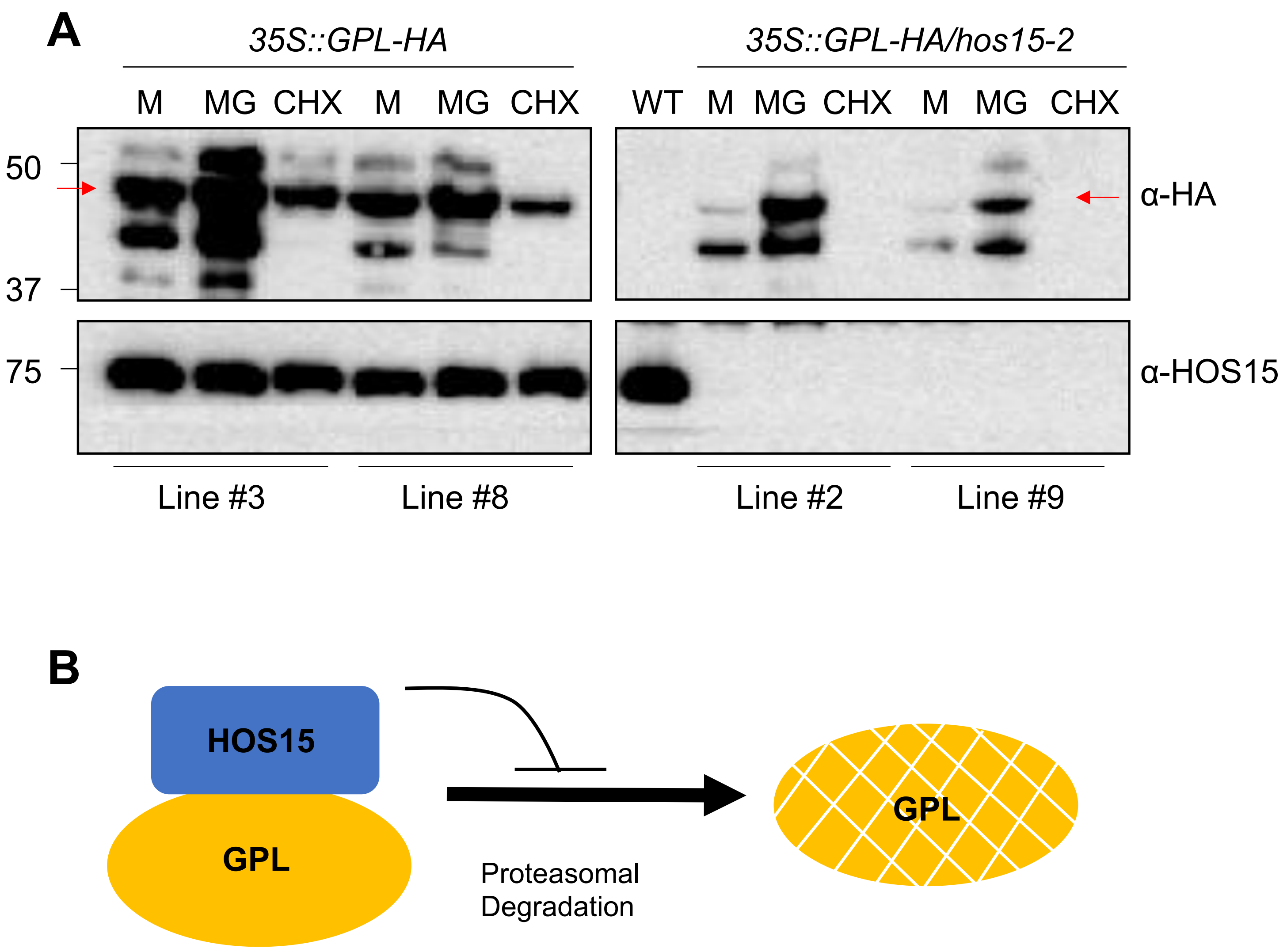

Figure S8. GPL stability is dependent on functional HOS15. (A) HOS15 is required for GPL stability. GPL is degraded in a 26s proteasome-dependent manner. Total proteins were extracted from 10-day-old transgenic lines expressing *35S::GPL-HA* in WT and *hos15-2* background (two independent stable lines from each genotypes, #3/#8 in WT and #2/#9 in *hos15-2* background) and treated with only MS media as Mock (M), proteasome inhibitor MG132 (MG) or cycloheximide (CHX), protein synthesis inhibitor. Immunoblots were performed using anti-HA and anti-HOS15 antibodies. Red arrows indicate GPL. **(B)** Presumed model illustrating that HOS15 protects GPL from proteasomal degradation.

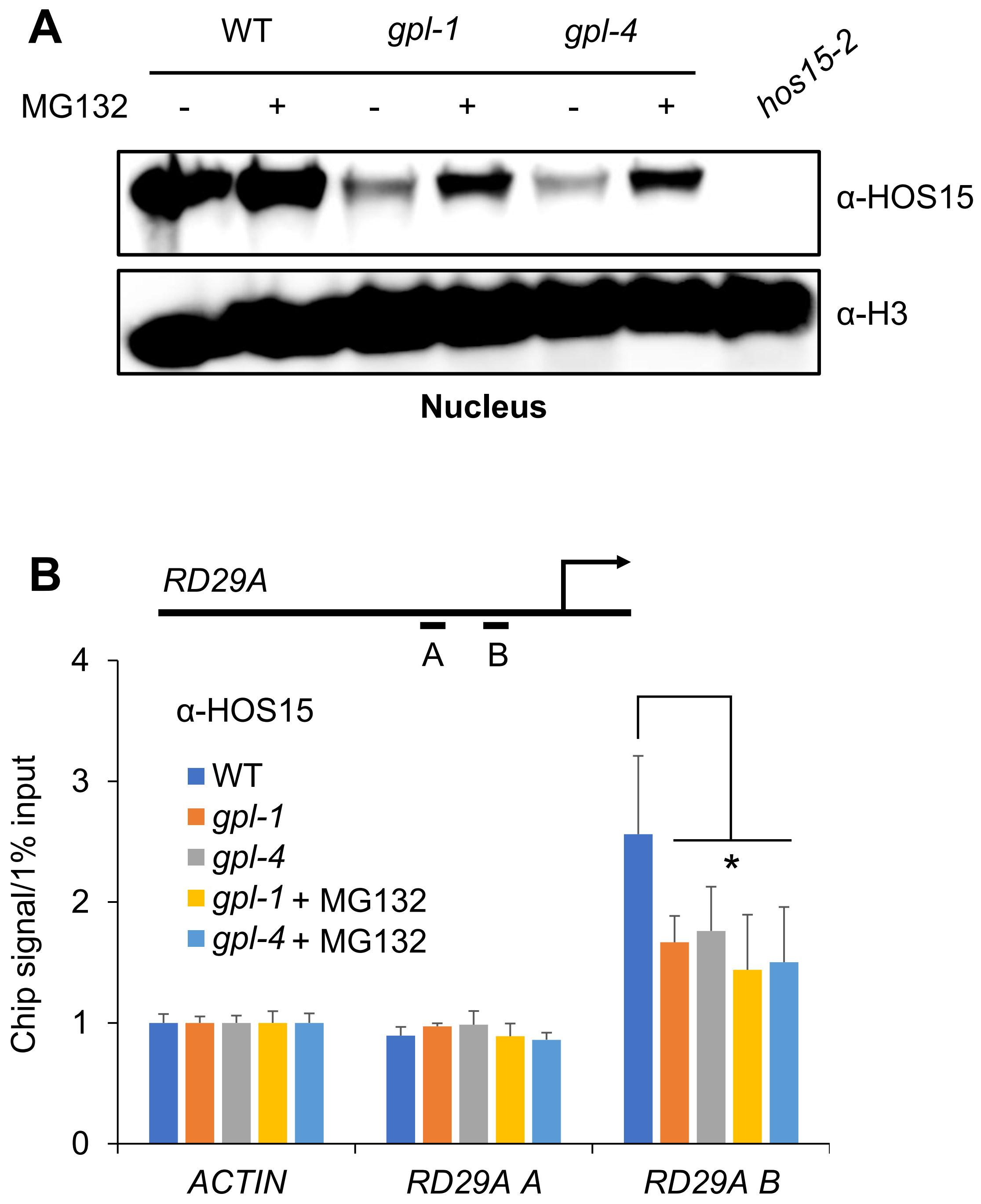

Figure S9. Functional GPL is required for HOS15 stability and activity. (A) Total proteins were extracted from 10-day-old seedlings of WT (Col-0) and *gpl*-mutants treated without or with proteasome inhibitor (MG132) for 4 h. Immunoblot were performed using anti-HOS15 antibodies. Histone3 (H3) was used as nuclear loading control. **(B)** HOS15 association with the target loci was reduced in *gpl*-mutants. *gpl*-mutants were treated with MG132 for 4 h to restore HOS15 protein levels to those observed in wild-type plants. Ch-IP assay was carried out using Anti-HOS15 antibodies. Promoter regions of *RD29A* were analyzed by ChIP-qPCR. ACTIN2 was used as internal control. Error bars represent SE. Significant difference was determined by a student's t-test (*p < 0.05).

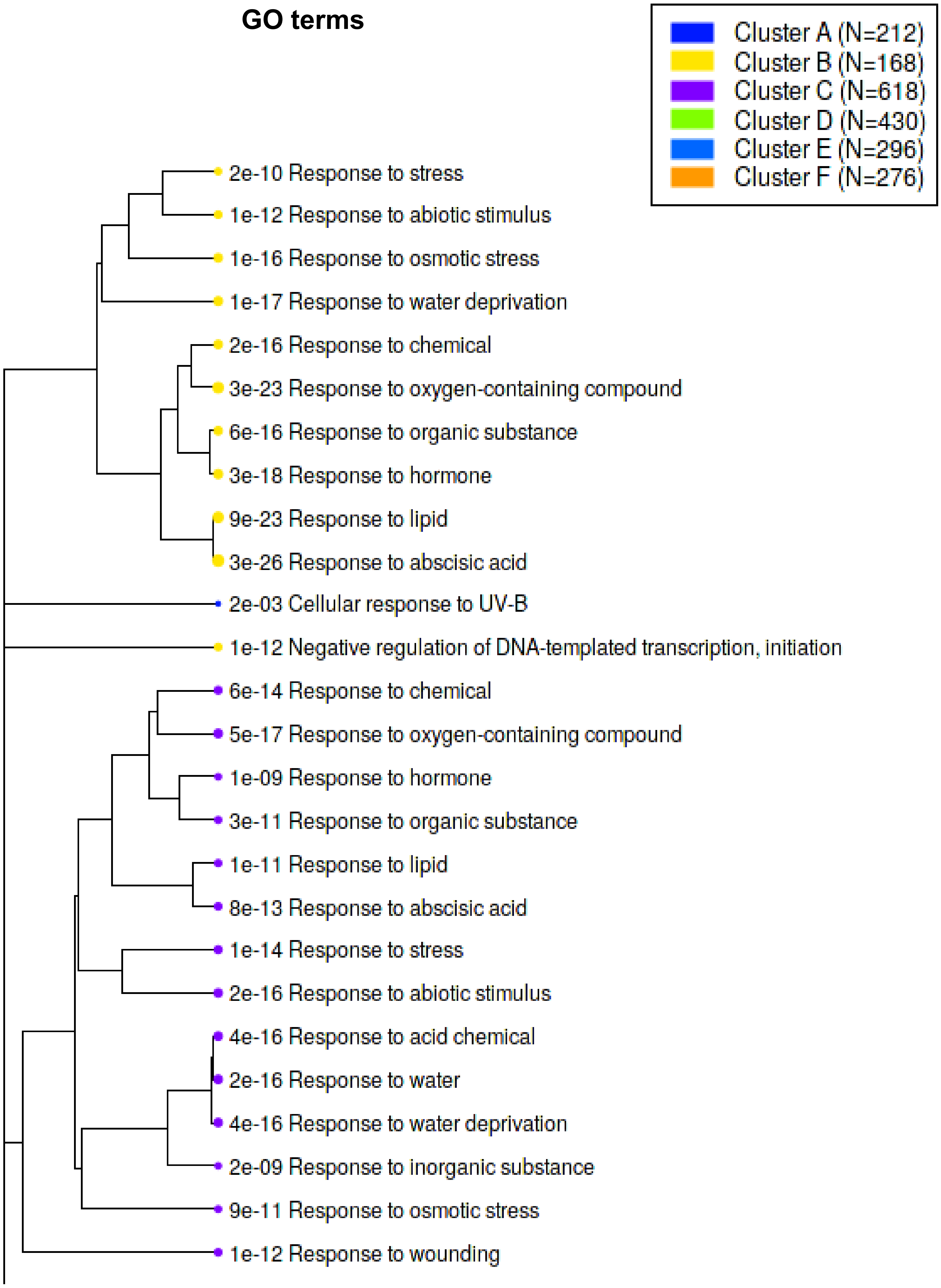
GO terms
Cluster A (N=212)
Cluster B (N=168)
Cluster C (N=618)
Cluster D (N=430)
Cluster E (N=296)
Cluster F (N=276)
2e-10 Response to stress
1e-12 Response to abiotic stimulus
1e-16 Response to osmotic stress
1e-17 Response to water deprivation
2e-16 Response to chemical
3e-23 Response to oxygen-containing compound
6e-16 Response to organic substance
3e-18 Response to hormone
9e-23 Response to lipid
3e-26 Response to abscisic acid
2e-03 Cellular response to UV-B
1e-12 Negative regulation of DNA-templated transcription, initiation
6e-14 Response to chemical
5e-17 Response to oxygen-containing compound
1e-09 Response to hormone
3e-11 Response to organic substance
1e-11 Response to lipid
8e-13 Response to abscisic acid
1e-14 Response to stress
2e-16 Response to abiotic stimulus
4e-16 Response to acid chemical
2e-16 Response to water
4e-16 Response to water deprivation
2e-09 Response to inorganic substance
9e-11 Response to osmotic stress
1e-12 Response to wounding

Continue….

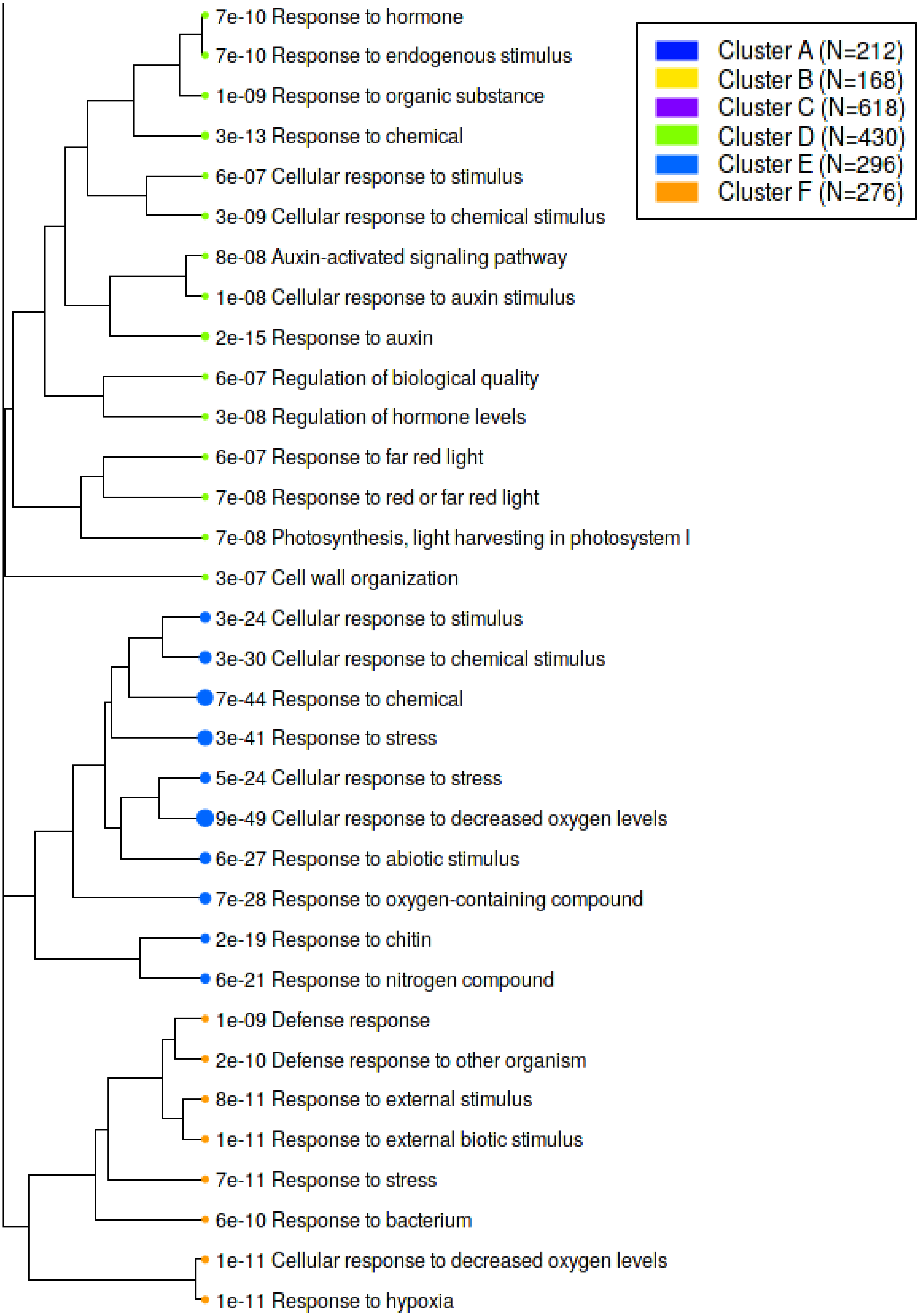

Figure S10. GO terms analysis of GPL-regulated genes (Clusters A-F).. As shown in Figure 4A (left border), global RNA-seq has been divided into six different clusters (A~F) as shown by different colors. Cluster A, Cluster B, Cluster C and Cluster E show upregulated genes (red color) which mostly belong to abiotic stress responses, whereas Cluster D and Cluster F show downregulated genes which belong to auxin signaling, growth and development and biotic responses. The dendrogram groups enriched GO biological process terms based on semantic similarity, highlighting redundancy among related terms. Numbers next to each term indicate the enrichment significance (FDR).

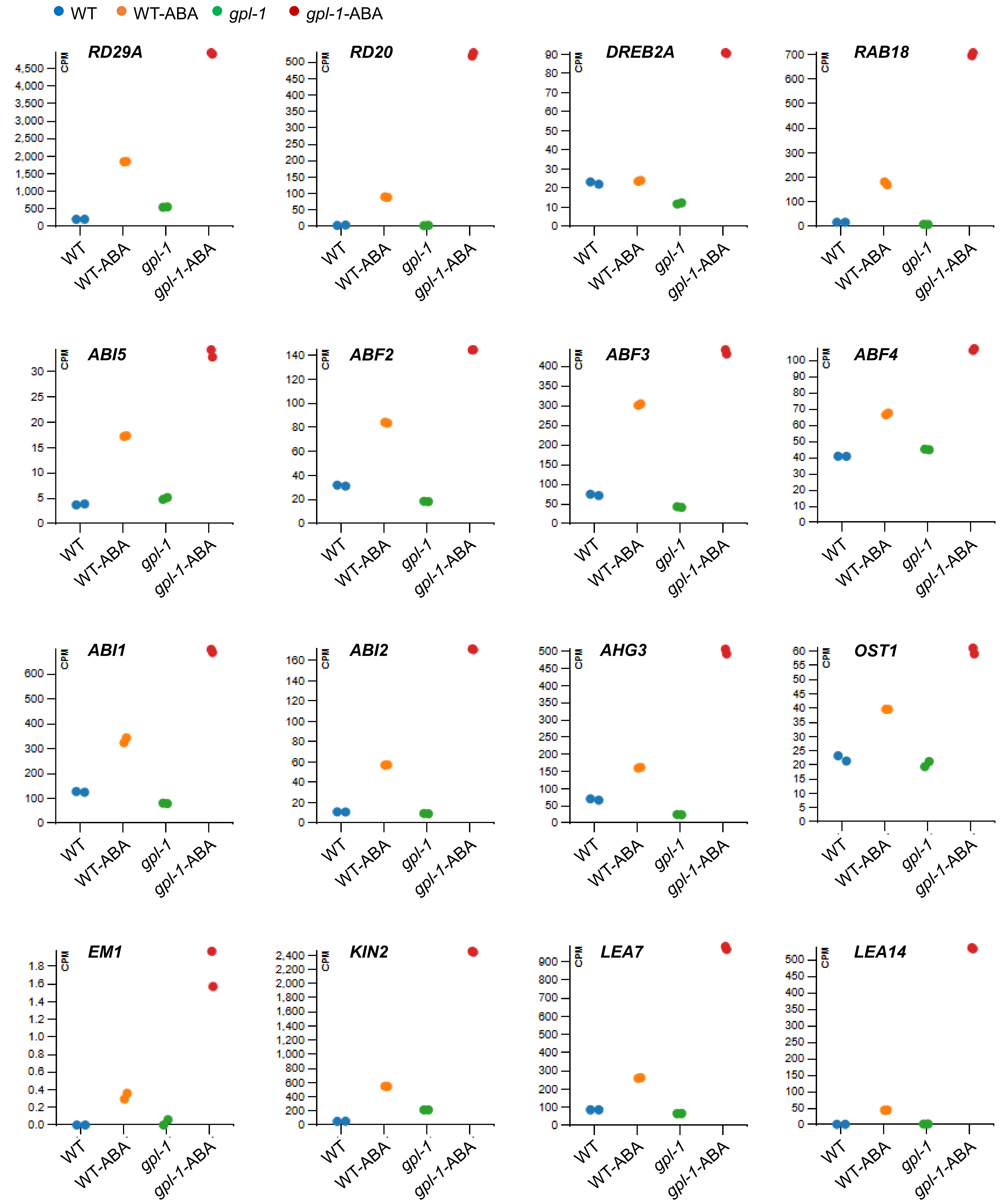

Figure S11. ABA related genes are strongly upregulated in *gpl-1*. Expression pattern of ABA responsive genes in *gpl-1* mutant (RNA-seq) which shows strong induction in *gpl*-mutant. Degust site (https://degust.erc.monash.edu/) was used to analyze the expression of ABA related genes in the RNA-seq. For further information see the link below; https://degust.erc.monash.edu/degust/compare.html?code=4d89de1d2f48529aaab30f868b178a8d#/

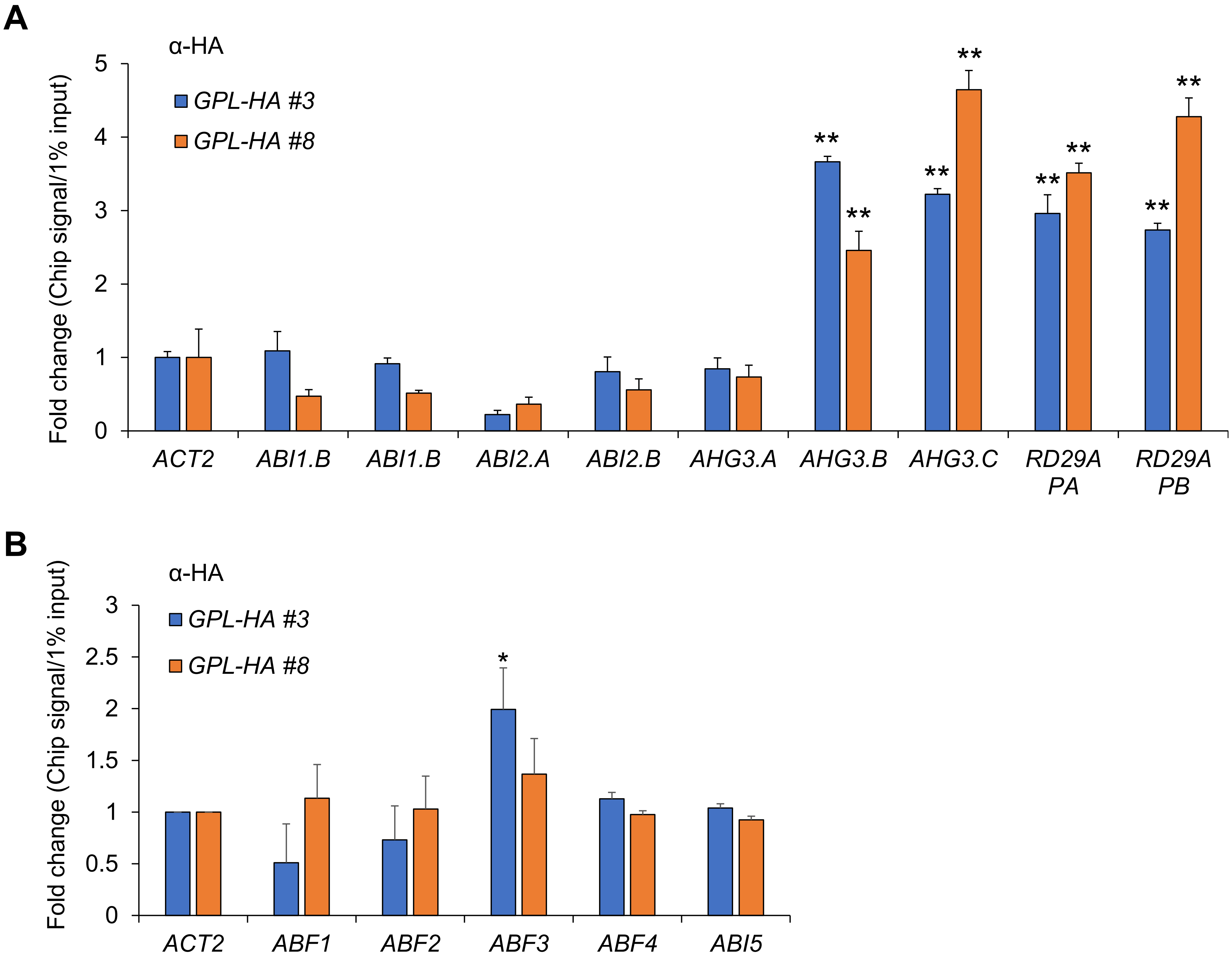

Figure S12. Association of GPL with the promoters of ABA responsive genes. (A) Association of GPL with the promoters of ABA responsive genes ABI1, ABI2 AHG3 and RD29A. Promoter regions of indicated genes were analyzed using ChIP-qPCR. Ch-IP assay was carried out using Anti-HA antibodies. ACTIN2 was used as internal control. Error bars represent SE. Significant difference was determined by student's t-test (**p < 0.01). **(B)** GPL does not associate with the promoters of ABF transcription factors. Promoter regions of *ABF1*, *ABF2*, *ABF3*, *ABF4* and *ABI5* were analyzed using ChIP-qPCR. Ch-IP assay was carried out using Anti-HA antibodies. ACTIN2 was used as internal control. Error bars represent SE. Significant difference was determined by student's t-test (*p < 0.05).

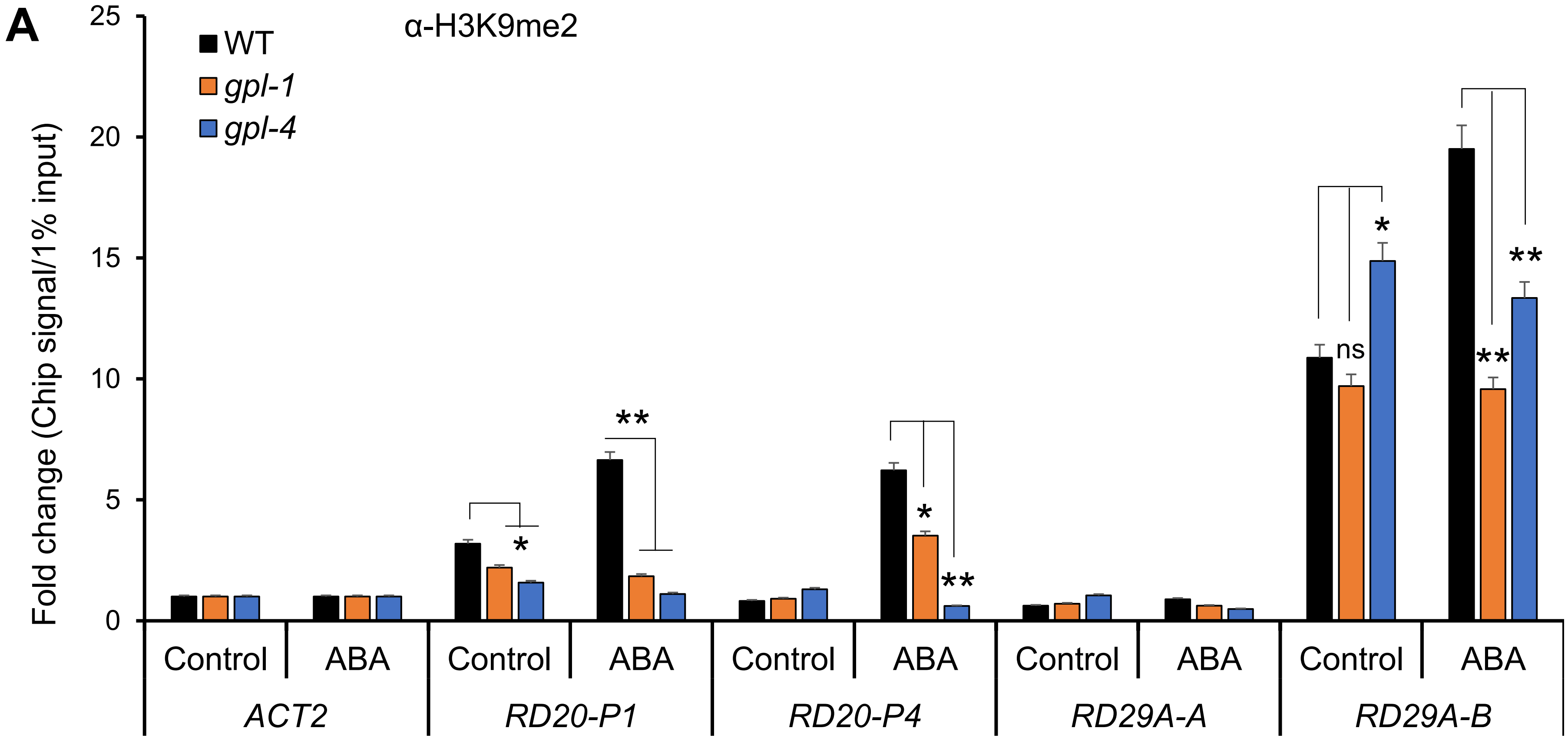

Figure S13. GPL regulates H3K9me2 at the promoters of ABA responsive genes. *GPL* mutation leads to an increase in H3K9 dimethylation at the promoter of *AHG3*. Promoter regions of indicated genes were analyzed using ChIP-qPCR. ACTIN was used as internal control. Error bars represent SE.

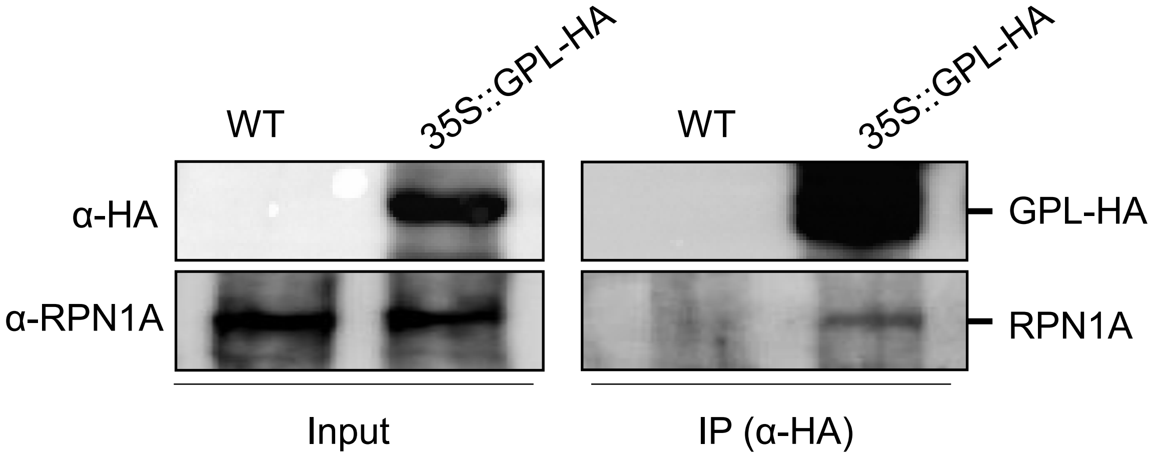

Figure S14. GPL interacts with RPN1A in Arabidopsis. Total proteins were extracted from 10-day-old WT and transgenic lines expressing *35S::GPL-HA* and immunoprecipitated with anti-HA antibodies. Immunoblots were carried out using anti-HA and anti-RPN1A.

Supplementary Table 1. GPL homolog from plant species.

Scientific Name	Max Score	Total Score	Query Cover	E-value	Percentage of identity	Accession length	Accession
Camelina sativa	122	122	93%	9.00E-34	88.06%	352	XP_010503395.1
Brassica rapa	94	94	91%	4.00E-23	75.76%	332	XP_009149865.1
Glycine max	54.3	54.3	63%	1.00E-08	68.09%	352	XP_003528169.2
Theobroma cacao	73.9	73.9	81%	1.00E-15	64.41%	346	XP_007099740.2
Gossypium hirsutum	70.9	70.9	77%	1.00E-14	67.86%	343	XP_016703400.2
Citrus sinensis	53.5	53.5	61%	2.00E-08	63.64%	364	XP_006476707.2
Solanum tuberosum	73.2	73.2	76%	2.00E-15	70.91%	326	KAH0659339.1
Solanum lycopersicum	71.6	71.6	76%	7.00E-15	70.91%	326	NP_001294882.1
Vitis vinifera	80.1	80.1	97%	7.00E-18	62.86%	361	XP_010663430.1
Triticum aestivum	51.6	51.6	59%	7.00E-08	53.49%	189	KAF7044309.1
Oryza sativa (Japonica)	52.8	52.8	73%	5.00E-08	52.83%	351	XP_015639948.1
Amborella trichopoda	52.4	52.4	59%	3.00E-08	55.81%	166	ERN01206.1
Physcomitrium patens	37.4	37.4	50%	0.013	47.22%	708	XP_024378696.1
Zea mays	53.1	53.1	58%	2.00E-08	54.76%	219	ONM07967.1

Supplementary Table 2. GPL-interacting protein identified through IP-MS.

No.	TAIR ID	NAME	Molecular Function
1	AT3G47850	GPL	Transcription co-regulator/ chromatin remodeling
2	AT4G67320	HOS15	WD40-repeat protein/ chromatin remodeling
3	AT3G52250	PWR	SAINT domain containing protein/ chromatin remodeling
4	AT3G44680	HDA9	Histone deacetylase
5	AT5G63110	HDA6	Histone deacetylase
6	AT2G20580	RPN1A	26S Proteasome regulatory subunit S2 1A
7	AT1G77080	AGL27/MAF1/FLM	MADS-box transcription factor
8	AT1G58110	bZIP transcription factor	bZIP transcription factor
9	AT2G36530	ENO2/LOS2	Enolase transcriptional activator; Multifunctional enzyme
10	AT5G04290	KTF1/SPT5L	kow domain-containing transcription factor 1
11	AT2G16485	NERD	DNA methylation; DNA binding/ zinc ion binding
12	AT5G40490	RNA-binding protein	RNA-binding (RRM/RBD/RNP motifs) family protein
13	AT4G09000	GRF1/GF14 CHI	General regulatory factor1-G-box factor 14-3-3
14	AT5G10450	GRF6/AFT1/14-3-3λ	G-box regulating factor 6
15	AT2G42590	GRF9/GF14 MU	General regulatory factor 9
16	AT1G22300	GRF10	14-3-3 General regulatory factor 10
17	AT5G14040	PHT3;1	Phosphate transporter 3;1
18	AT4G35100	PIP3/PIP3A/PIP2;7/ SIMIP	Plasma membrane intrinsic protein 3
19	AT5G15090	VDAC3/ATVDAC3	Voltage dependent anion channel 3
20	AT1G12840	DET3/ATVHA-C	Vacuolar ATP synthase subunit C

Supplementary Table 3. Primer sequences.

Name	Sequence	Purpose
**Cloning**		
GPL-attB1	AAAAAAGCAGGCTTCATGCAAGTCGAAATCCCATCG	cloning
GPL-attB2	AGAAAGCTGGGTCCTAATTCCATGGACTCTGAAG	
GPL-F-SacII	tgCCGCGGtATGCAAGTCGAAATCCCATCG	cloning
GPL-R-SpeI	cACTAGTATTCCATGGACTCTGAAGCCG	
sgRNA1-F	GATTGGTGGCGATATCTATGTACAG	CRISPR
sgRNA1-R	AAACCTGTACATAGATATCGCCACC	
sgRNA2-F	GATTGGTTGGTACTCACATGGAAGG	CRISPR
sgRNA2-R	AAACCCTTCCATGTGAGTACCAACC	
HOS15 attB1	AAAAAAGCAGGCTTAATGTCTTCACTTACCTCCGTCG	cloning
HOS15 attB2	AGAAAGCTGGGTGCTACATTCTGAAATCAAGAACG	
HDA9 attB1	AAAAAAGCAGGCTTAATGCGTTCCAAGGACAAAATCTC	cloning
HDA9 attB2	AGAAAGCTGGGTCTTATGACGCATCGTTATCGTTGTC	
PWR attB1	AAAAAAGCAGGCTTCATGCCGCAGGATCACGCTTCGTGG	cloning
PWR attB2	AGAAAGCTGGGTCTCACGTGGCTGCCTCTGCTACACCA	
**Genotyping**		
GPL-FL-F	ATGCAAGTCGAAATCCCATCG	genotyping
GPL-FL-R	ATTCCATGGACTCTGAAGCCG	
**qRT-PCR**		
UBQ5-qPCR-F	GACGCTTCATCTCGTCC	qRT-PCR
UBQ5-qPCR-R	GTAAACGTAGGTGAGTCC	
LEA7-qPCR-F	GCAATCAAGAACAAGGCACA	qRT-PCR
LEA7-qPCR-R	TCAGTGCGAAGCCCTAAAGT	
DREB2A-qPCR-F	CAGTGTTGCCAACGGTTCAT	qRT-PCR
DREB2A-qPCR-R	AAACGGAGGTATTCCGTAGTTGAG	
RD20-qPCR-F	TCACCATTATTGCCGGTTTA	qRT-PCR
RD20-qPCR-R	ATTTCCCTCGGTTACATTCC	
RD29A-qPCR-F	ATCACTTGGCTCCACTGTTGTTC	qRT-PCR
RD29A-qPCR-R	KACAAAACACACATAAACATCCAAGT	
AHG3-qPCR-F	CGACGTTACCGGGAAATCTAG	qRT-PCR
AHG3-qPCR-R	TAGCCTTCTTATTCTCACTCCTCAC	
**ChIP-qRT-PCR**		
ACTIN2-F	CGTTTCGCTTTCCTTAGTGTTA	ChIP-qRT
ACTIN2-R	AGCGAACGGATCTAGAGACTC	
ABI1-A-F	GATATTTTACCGGTGGTC	ChIP-qRT
ABI1-A-R	GACGTGTCGTAGTCCGAGTT	
ABI1-B-F	CTTGTCTTCCTAGCTTCTTC	ChIP-qRT
ABI1-B-R	CCTTTACCCAATCTGATCCC	
ABI2-A-F	CTAGTGTGGTCAGTGTAGATG	ChIP-qRT
ABI2-A-R	GTGTAACATGCCATATGTCAC	
ABI2-B-F	CTCTCCTTTCTCTTCCCAAC	ChIP-qRT
ABI2-B-R	GAGGGTCAGTGAATGGTCTG	
AHG3-A-F	TGTAACCCTTCCATGCGAAAA	ChIP-qRT
AHG3-A-R	AAGTTGAAGAAGAACGCAAAA	
AHG3-B-F	GCTGACTCCGCCGTTGCTCCT	ChIP-qRT
AHG3-B-R	TGTCTCACGCTTCTGCCGTTT	
AHG3-C-F	TAAAGAAGTTAGCCAACGAGA	ChIP-qRT
AHG3-C-R	TACTCCGAGTAGCACCATTAA	
RD29A-A-F	CATTTTAGGATGGAATAAATATCAT	ChIP-qRT
RD29A-A-F	GCTTTTTGGAACTCATGTCGGTAGT	
RD29A-B-F	TACAATTCGAATGAGAAGGATGTGC	ChIP-qRT
RD29A-B-F	TAACTTACTATTATTAGTAGTCGGT	
RD20-P1-F	GTTCTTGCAAACAACTACGG	ChIP-qRT
RD20-P1-R	GCAAAACATGATTGACTGGG	
RD20-P4-F	CGAAAAGTACGGAACGATTT	ChIP-qRT
RD20-P4-R	TAACCACTTAATTTCCCGCT	
ABF1.F	TCC TAA CTC ATC TGA AGT TTA TG	ChIP-qRT
ABF1.R	TCC TAA CTC ATC TGA AGT TTA TG	
ABF2.F	CTG ATT CTT TTG CAT TTC CAT G	ChIP-qRT
ABF2.R	TTA CAA CGA AAG CAG GTA ATC AC	
ABF3.F	TTA ACC GTT CTC AAC CTG CAA C	ChIP-qRT
ABF3.R	CAT ACA TGC ATA TCC AAA ACC AGC	
ABF4.F	GGT AGG AGT AAA GAA CAC TGT C	ChIP-qRT
ABF4.R	GAT ATC ATC AGT ATT GTT TAG ACA C	
